# Artificial Intelligence and Automation in Evidence Synthesis: An Investigation of Methods Employed in Cochrane, Campbell Collaboration, and Environmental Evidence Reviews

**DOI:** 10.1002/cesm.70046

**Published:** 2025-08-28

**Authors:** Kristen L. Scotti, Sarah Young, Melanie A. Gainey, Haoyong Lan

**Affiliations:** ^1^ Carnegie Mellon University Libraries Carnegie Mellon University Pittsburgh Pennsylvania USA

**Keywords:** artificial intelligence, living reviews, machine learning, screening automation, systematic reviews

## Abstract

Automation, including Machine Learning (ML), is increasingly being explored to reduce the time and effort involved in evidence syntheses, yet its adoption and reporting practices remain under‐examined across disciplines (e.g., health sciences, education, and policy). This review assesses the use of automation, including ML‐based techniques, in 2271 evidence syntheses published between 2017 and 2024 in the *Cochrane Database of Systematic Reviews*, and the journals *Campbell Systematic Reviews*, and *Environmental Evidence*. We focus on automation across four review steps: search, screening, data extraction, and analysis/synthesis. We systematically identified eligible studies from the three sources and developed a classification system to distinguish between manual, rules‐based, ML‐enabled, and ML‐embedded tools. We then extracted data on tool use, ML integration, reporting practices, motivations for (and against) ML adoption, and the application of stopping criteria for ML‐assisted screening. Only ~5% of studies explicitly reported using ML, with most applications limited to screening tasks. Although ~12% employed ML‐enabled tools, ~90% of those did not clarify whether ML functionalities were actually utilized. Living reviews showed higher relative ML integration (~15%), but overall uptake remains limited. Previous work has shown that common barriers to broader adoption included limited guidance, low user awareness, and concerns over reliability. Despite ML's potential to streamline evidence syntheses, its integration remains limited and inconsistently reported. Improved transparency, clearer reporting standards, and greater user training are needed to support responsible adoption. As the research literature grows, automation will become increasingly essential—but only if challenges in usability, reproducibility, and trust are addressed.

## Graphical abstract

Machine learning and automation remain underutilized and inconsistently reported in evidence syntheses. Our large‐scale review of 2,271 studies highlights limited adoption beyond screening, with significant gaps in transparency, reporting, and responsible implementation—underscoring the urgent need for clearer guidelines and better integration of AI tools in systematic review workflows.

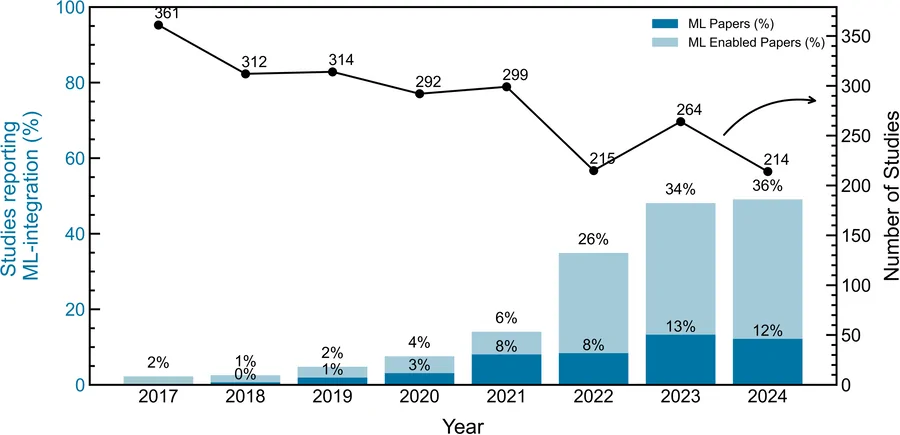

## 1 Introduction

Evidence synthesis involves systematically aggregating and, in some cases, evaluating research findings to expand their applicability and generate new knowledge [1, 2, 3, 4]. It plays a critical role in advancing scientific knowledge by building consensus [5, 6], informing evidence‐based practices [7, 8, 9], identifying knowledge gaps [10, 11], and guiding policy [12, 13]. Across disciplines, evidence synthesis methods (e.g., systematic reviews [14], meta‐analyses [5], and evidence and gap maps [10]) are used to address complex questions and support decision‐making. For example, in medicine, evidence synthesis is integral to the development and evaluation of clinical guidelines [7]; it also helps to inform best practices in education [15] and environmental policy [13]. Challenges in replicating research findings across disciplines has underscored the need for rigorous evidence synthesis methods as a mechanism for identifying sources of variability in results and promoting the reliability of research [5, 16].

Preparing an evidence synthesis involves a series of structured steps [17]. First, the research team formulates a research question using a framework such as PICO [18] and develops a protocol outlining the research plan. Then they conduct systematic and comprehensive searches of bibliographic databases, trial registers, and other sources of published and unpublished literature. At least two team members independently select studies for inclusion in two stages using predefined eligibility criteria: first through title and abstract screening, followed by full‐text screening of any studies not excluded during the title and abstract screening stage. Citation searching of the included studies is also performed to find any missing studies. At least two independent team members assess the included studies for risk of bias and extract data from the included studies. Finally, the extracted data are synthesized and summarized. Some types of evidence synthesis, such as scoping reviews and evidence and gap maps, typically do not include a risk of bias assessment [14].

Reporting standards are structured guidelines that promote transparency and consistency in documenting evidence synthesis methods, helping ensure reproducibility and supporting evaluation by others [19]. The most commonly used reporting standards for evidence synthesis include the Preferred Reporting Items for Systematic reviews and Meta‐Analysis (PRISMA) [20] and its associated extensions, Campbell Collaboration's newly revised Methodological Expectations of Campbell Collaboration Intervention Reviews (MECCIR) [21], and the Reporting Standards for Systematic Evidence Syntheses (ROSES) from the Collaboration for Environmental Evidence [22].

Evidence synthesis demands significant human effort, often requiring teams of experts months or even years to complete [23, 24]. These efforts are compounded by the exponential growth of research outputs [25, 26], which makes comprehensive synthesis tasks increasingly challenging. Reducing the time and effort required for evidence synthesis, while maintaining methodological rigor, is essential for delivering timely insights to stakeholders and decision‐makers.

While previous reviews [27, 28, 29, 30, 31, 32, 33, 34, 35, 36, 37] have examined machine learning (ML)‐based automation in evidence synthesis, these studies have largely focused on specific review stages (e.g., automation in screening [38, 39], data extraction [30]). We are not aware of any work to date that has simultaneously quantified both the extent of ML adoption and the transparency of its reporting across all evidence synthesis stages (search, screening, data extraction, synthesis) and across three leading review venues (Cochrane, Campbell Collaboration, and Environmental Evidence). Our analysis of 2271 syntheses makes three novel contributions:

1. *Comprehensive scope*. We chart year‑by‑year uptake of manual, rules‑based, ML‑enabled, and ML‑embedded tools across Cochrane, Campbell, and Environmental Evidence.
2. *Reporting audit*. We document where authors omit important reporting details, such as stopping rules, classifier validation, and translation methods, and explain how these gaps hinder reproducibility.
3. *Cross‑discipline insight*. We document and compare ML adoption rates and reporting patterns across reviews from health (Cochrane), social and behavioral sciences (Campbell Collaboration), and environmental science (Environmental Evidence), highlighting significant areas sources differ while noting our sample imbalance.

Additionally, we took a retrospective approach to our examination, beginning in 2017, well before the emergence of large language models (LLMs) like ChatGPT [40]. This timeframe was selected to capture both early uses of ML and more recent developments, offering a fuller picture of the trajectory of automation adoption.

The paper is organized as follows: we first review existing automation tools and reporting standards, then describe our methods (Section 2), present adoption trends and reporting gaps (Section 3), discuss implications for guideline development (Section 4), and conclude with recommendations for future tool design and reporting (Sections 4 and 5).

### 1.1 Early Automation Applications in Evidence Synthesis

Before the widespread use of digital databases, evidence synthesis processes often involved physically searching through library archives, scanning printed abstracts in conference proceedings, or using index cards to track relevant studies. Screening, data extraction, and synthesis processes were similarly laborious [41]. As digital technology has advanced, automation has become an integral part of improving efficiency in these processes [28, 31, 32, 36, 37]. Early efficiency improvements relied on deterministic methods, using predefined rules to process information and identify relationships. For example, Boolean search strategies refine literature searches by applying logical operators (e.g., AND, OR, NOT) [42]. Similarly, basic text‐mining techniques that filter results based on word frequency or other simple heuristics use deterministic methods to improve efficiency in study selection [43]. More advanced deterministic approaches, such as ontology‐driven reasoning, rely on structured systems of knowledge to help organize and relate information. Instead of just matching keywords, these approaches use predefined relationships between concepts to improve how studies are grouped and categorized. For example, biomedical ontologies like MeSH (Medical Subject Headings) or the Unified Medical Language System (UMLS) help expand search results by linking related terms [44, 45].

The growing prevalence and accessibility of artificial intelligence has introduced new opportunities for automating evidence synthesis tasks [30, 32, 36, 46]. Artificial Intelligence (AI) broadly refers to computer systems designed to perform tasks that typically require human intelligence, such as reasoning, problem‐solving, and pattern recognition [47]. While early AI systems relied on rules‐based methods, modern AI increasingly incorporates ML techniques. ML is a subset of AI that enables systems to learn from data and make predictions or classifications without relying solely on predefined rules [48].

### 1.2 Current ML Applications in Evidence Synthesis

ML techniques have been increasingly applied to reduce the time and effort required to complete evidence synthesis tasks, most often in the screening phase [32, 46, 49, 50, 51]. In ML‐assisted screening, algorithms are used to analyze titles and abstracts to predict which records are most likely to be relevant. In some cases, these algorithms are used to rank studies such that abstracts that are more likely to meet inclusion criteria are screened first. In others, studies predicted to be irrelevant are automatically excluded from screening [52, 53]. Active learning, the most commonly employed approach used in ML‐assisted screening [36, 54, 55, 56], works iteratively. The model selects subsets of studies for human review and continuously refines its predictions based on human feedback. A limitation of active learning is its sensitivity to initial training data, which may introduce bias or delay model convergence if the early labeled examples are unrepresentative [57, 58]. Supervised learning techniques are also used; these models are trained on data previously labeled by human experts (abstracts that have been categorized as relevant or irrelevant). Among supervised techniques, Support Vector Machines (SVMs) classify studies by identifying textual boundaries that distinguish “relevant” versus “irrelevant,” abstracts based on textual features (e.g., the presence of key terms or patterns in the text) [34, 59]. These SVM models can handle large volumes of text, but their reliance on human‐labeled data limits their ability to capture complex language patterns.

Deep learning approaches improve the models' ability to identify more nuanced text patterns. For example, sequential text processing by Recurrent Neural Networks (RNNs) enables models to process input word‐by‐word while maintaining a form of memory through internal hidden states [60, 61]. This helps capture dependencies across a sequence of text, allowing the model to interpret meaning based on context and word order, making them useful for analyzing abstracts where earlier terms influence the interpretation of later ones. Originally developed for image recognition applications, Convolutional Neural Networks (CNNs) analyze sequences of words or characters by detecting recurring patterns (e.g., co‐occurring phrases or linguistic structures [31, 46]). Lastly, transformer models, such as BERT (Bidirectional Encoder Representations from Transformers), analyze entire sentences simultaneously, improving efficiency and accuracy by capturing deeper contextual relationships between words [32, 50]. However, deep learning models often require large labeled data sets and substantial computational resources, posing scalability and reproducibility challenges for evidence synthesis applications [62].

While screening has been the primary focus of ML applications in evidence synthesis, researchers have also explored ML techniques for data extraction (the process of identifying, extracting, and organizing key study details from included studies). Natural Language Processing (NLP) methods, such as Named Entity Recognition (NER), label specific information types like patient groups or treatment names from unstructured text [30, 35, 46]. Other ML approaches classify or extract structured study elements, like study results or methodological details. Early methods relied on supervised learning to identify similar elements in new texts [30], while newer methods, such as transformer‐based models like BERT, have improved automated data extraction by considering context and complex language structures, rather than isolated keywords or phrases [34, 50]. However, fully automated data extraction remains challenging due to inconsistent reporting across publications. Hybrid approaches that combine ML with rule‐based methods and human oversight are often required to ensure accuracy and reliability in data extraction [30, 31].

ML‐based automation techniques for analyzing and synthesizing findings have received less attention relative to automation in screening and data extraction. Techniques like topic modeling offer promise for identifying themes across a body of literature. For example, Latent Dirichlet Allocation (LDA) is a topic modeling technique that analyzes word distributions within studies to identify clusters of related topics. By grouping studies based on shared concepts, topic modeling can provide a high‐level overview of research trends and connections [32].

Integrating ML into evidence synthesis significantly reduces time and effort [36, 63, 64]. However, challenges associated with a lack of established guidelines for implementing ML [32, 46], ensuring reproducibility of ML practices [36, 50, 65], assessing performance of ML‐enabled tools [46, 51], and addressing biases inherent in algorithmic systems [66], impede broader adoption. Inconsistencies in documenting the application of these technologies complicate evaluations of their effectiveness and applicability [67]. The recently published Digital Evidence Synthesis Tools for Climate and Health report [68] investigated the use of automation tools in evidence synthesis workflows by analyzing methodologies from published reviews and assessing user perspectives. The researchers found that, while the number of evidence synthesis automation tools has grown, their uptake has been limited, with only ~56% of reviewed studies reporting their use. The report identified barriers to researcher adoption of automation tools, including a lack of formal evaluations and guidelines, concerns about reliability and methodological rigor, and misconceptions about the complexity of these tools.

### 1.3 Evolution of Reporting Standards in the Context of AI

As artificial intelligence and ML are increasingly applied within evidence synthesis workflows, reporting standards play a critical role in addressing related challenges. These challenges include ensuring transparency around how AI/ML tools are used, supporting reproducibility of results, and enabling proper evaluation of tool performance. To meet these needs, reporting standards have begun evolving to include more explicit guidance on documenting automation methods and AI applications. Among these standards, PRISMA, updated in 2020 [20], provides the most detailed recommendations for reporting the use of AI/ML. These can be found in the expanded checklist and are largely focused on the use of AI/ML in screening. The standard recommends that the use of ML classifiers for screening be reported including information about the software or classifier, classifier version, how the classifier was used and trained, and whether any internal or external validation was carried out. It also recommends that the use of ML for prioritized screening be reported including the software and details of screening rules. Beyond screening, PRISMA recommends reporting the use of “automation tools” for search strategy translation and the use of NLP or text frequency analysis tools for search term identification. It notes that automation tools used for data extraction, risk of bias assessment and certainty assessment should be reported along with details about how they were used, trained and validated. If abstracts or articles are translated into other languages, the process for doing so should be described.

The recently updated Campbell Collaboration reporting standard [69] acknowledges the potential use of AI/ML in the screening step of the review process, stating “If automation is used (e.g., ML. AI screening of title and abstract), describe how, which software, including any validation (e.g., 10% review by human), if used.” ML is also mentioned in respect to data extraction, but with little detail provided.

ROSES, from the Collaboration for Environmental Evidence [22], most recently updated in 2017, mentions the importance of transparency in methods, though explicit references to AI/ML are limited. Similarly, the Cochrane Handbook [70] describes various types of automation in the evidence synthesis process but provides minimal guidance on how best to report the use of these tools. Cochrane retired its reporting standards in 2023 and now endorses PRISMA [21].

Table S1 summarizes the current coverage of these key reporting standards in respect to the use of automation, ML and AI tools and methods.

### 1.4 Study Aim

This study aims to analyze evidence syntheses published in the *Cochrane Database of Systematic Reviews*, and the journals *Campbell Systematic Reviews*, and *Environmental Evidence* to assess the evolving use and reporting practices of automation in evidence synthesis. These sources were chosen because the organizations tend to adhere to relatively high methodological standards and cover a broad disciplinary spectrum. By examining trends and reporting practices, as well as identifying gaps in applying these techniques, this study provides a cross‐disciplinary snapshot of current practices and informs efforts to strengthen guidance and improve reporting to support the responsible integration of AI and ML into evidence synthesis workflows.

## 2 Methods

The methods for this review were preregistered in February 2025 [71].

### 2.1 Research Questions

This study aims to answer the following research questions:

- **RQ‐1.** How has the adoption of manual, automated, and ML‐based techniques in evidence synthesis evolved across key review stages (search, screening, data extraction, and analysis)?
- **RQ‐2.** What types of automation and ML‐based techniques are currently employed at each review stage, and to what extent are their implementation details transparently reported in published studies?
- **RQ‐3.** What justifications or motivations do researchers provide for their decisions to use or not use ML or other automation techniques in evidence synthesis?

### 2.2 Eligibility Criteria

This review includes all evidence syntheses published in the *Cochrane Database of Systematic Reviews*, and the journals *Campbell Systematic Reviews*, and *Environmental Evidence* between January 1, 2017, and December 31, 2024. The following types of publications were excluded: protocols, methods papers, editorials, commentaries, and updates to previous reviews. For updates to reviews, we determined the year of the original review. If the original review was published within the inclusion timeframe, it was included, and any subsequent updates were reviewed to assess any methodological changes in the review process.

The inclusion timeframe was determined through a preliminary review of studies published from 2000 forward, which indicated that ML techniques began to be employed in reviews published in *Cochrane Database of Systematic Reviews*, *Campbell Systematic Reviews*, and *Environmental Evidence* around 2017. Moreover, the most employed ML technique identified in the preliminary review was the RCT classifier, a ML algorithm designed to identify randomized control trials. This tool was introduced by Cochrane in late 2018 [63, 72].

### 2.3 Search Strategy

We examined the following websites to identify relevant studies:

- *Cochrane Database of Systematic Reviews* (https://www.cochranelibrary.com/cdsr/reviews),
- Campbell Collaboration, *Campbell Systematic Reviews* (via Wiley: https://onlinelibrary.wiley.com/journal/18911803), and
- *Environmental Evidence Journal* (https://environmentalevidencejournal.biomedcentral.com/)

We conducted a title‐by‐title review of studies available on these websites that were published within the timeframe of January 1, 2017, to December 31, 2024. We then exported the bibliographic information for identified studies to Zotero for further assessment against the eligibility criteria described above.

### 2.4 Selection Process

Studies published within *Cochrane Database of Systematic Reviews*, and the journals *Campbell Systematic Reviews*, and *Environmental Evidence* are preclassified by publication type. Inclusion decisions were made based on these classifications. Due to the straightforward nature of the inclusion criteria, the search and selection process was conducted by a single reviewer (K. S.).

### 2.5 Data Extraction

A bibliographic file (RIS) of included studies was generated using Zotero and uploaded to a publicly available Sysrev [73] project (https://www.sysrev.com/o/1605/p/199195) for data management. Sysrev is a web‐based platform for collaborative evidence synthesis that supports article screening and structured data extraction [73]. We conducted an initial review of ~1500 studies to formulate a list of reported tool usage across the review stages of search, screening, data extraction, and analysis/synthesis. This tools list was used to create a data extraction form in Sysrev. The following data were extracted from each included study:

- Bibliographic details describing the study (auto‐extracted by Sysrev)
- Review type (as categorized by the journal),
- Reference manager (software used for managing references),
- Tools/software used for deduplication, citation searching, and/or language translation,
- Tools/software used during search, screening, data extraction, and analysis/synthesis (standard databases used for searches were not recorded),
- Any descriptions of the use of artificial intelligence tools/techniques, including ML, and
- The total number of records screened (after duplicate removal) and the number of records included for data extraction.

The tools list was also utilized to automate the highlighting of study PDFs imported into Sysrev. Included studies were tagged in Zotero for PDF processing and the Zotero API was accessed via Python to create a list of tagged articles. The Python script retrieved file paths for the PDF attachments, copied the PDFs into a processing folder, and applied highlights using the tool list as keywords. The highlighted PDFs were then uploaded to Sysrev using the batch upload feature, where Sysrev automatically matched the PDFs to the corresponding articles based on filenames. This automated highlighting process was designed to expedite the data extraction workflow.

Given the large number of included studies (over 2000), a single reviewer (K. S.) extracted data from all studies, while three additional reviewers (S. Y., M. G., H. L.) independently extracted data from a subset of the studies. Inter‐rater reliability (IRR) was assessed using Cohen's κ [74], a statistical measure of agreement between two raters that accounts for agreement occurring by chance. A benchmark kappa value of 0.81 was established based on conventional thresholds indicating “strong” agreement [75]. The review team met weekly to discuss variations in data extraction, and the total number of articles to undergo duplicate data extraction was determined by calculating kappa at regular intervals. Duplicate data extraction was intended to continue until a satisfactory κ value (0.81 or higher [75]) was consistently achieved. However, the initial κ calculation for the first subset of studies (49 studies) was ~0.90, exceeding the initial benchmark. As variations in data extraction were observed in the first subset, we extracted data in duplicate for a second set of studies (*n* = 64) to ensure consistency. The kappa for this second subset was ~0.92. In total, duplicate data extraction was conducted for 113 studies (~5% of the included studies).

### 2.6 Tool Classification and Definitions

To evaluate the extent and nature of automation in evidence synthesis workflows, all software and tools used in included studies were classified into four categories based on their level of automation and integration of ML. This classification enabled us to distinguish between tools that offer ML capabilities and those that depend on ML by design. The following classifications were used:

- *Manual*: Tools or workflows that involve no automation or ML capabilities. These typically include spreadsheets and standardized forms.
- *Automated* (rules‐based): Tools or workflows that apply predefined, deterministic rules or heuristics to perform tasks (e.g., Boolean search operators, automated deduplication based on metadata matching, or keyword‐based screening).
- *ML‐enabled*: Tools that integrate optional ML functionality. ML features may be used within the tool but can be disabled by the user (e.g., Covidence after 2022 [76, 77]). All tools that integrate optional ML functionality were classified as ML‐enabled irrespective of whether those features are enabled by default.
- *ML‐embedded*: Tools or workflows in which ML is a core, non‐optional component. These tools rely on ML to perform key functions, and ML cannot be disabled (e.g., Cochrane's RCT Classifier [63, 72, 78, 79] and Swift Active Screener [80]).

In cases where the included studies did not specify whether a tool used ML or what type of automation it involved, we sought external documentation—including developer websites, user manuals, published evaluations, and software release notes—to determine the most accurate classification. Classifications were based on the tool's capabilities as of the year it was reportedly used in the study. When major ML features were introduced mid‐study period (e.g., for Covidence [76, 77] and Rayyan [81]), the classification of that tool was updated accordingly for studies published after the documented release. A full list of tool classifications by task and study year is provided in Table S2.

For studies that explicitly reported the use of ML tools, this was recorded as a separate variable, independent of the tool classification, to capture intentional and reported use of ML in practice.

### 2.7 Data Analysis

Data analysis was performed in Python; raw data and the code used for analysis is available on the Open Science Framework project page (https://osf.io/gch5e/). Where appropriate, data are expressed as mean ± standard deviation.

## 3 Results

Throughout this paper, we use the term, “automation” to refer to any non‑manual computational assistance in evidence synthesis, including both rules‑based algorithms (e.g., Boolean search operators, deduplication heuristics) and ML methods. We use “artificial intelligence (AI)” interchangeably with automation, but reserve “ML” for those AI approaches that learn predictive or classification models from data.

### 3.1 Search Results

A total of 2271 evidence synthesis studies were included in our analysis. The majority (~89%, 2025 studies) were published in the *Cochrane Database of Systematic Reviews*, followed by the *Campbell Collaboration* (~8%, 161 studies), and the *Collaboration for Environmental Evidence* (~4%, 85 studies). Systematic reviews accounted for ~94% (2131 studies) of the total, while evidence gap maps comprised ~4% (82 studies). The data set also included 34 umbrella reviews (~1.5%), 18 rapid reviews (< 1%), and 6 scoping reviews (< 1%).

### 3.2 Evolution of Automation

**Figure 1:**
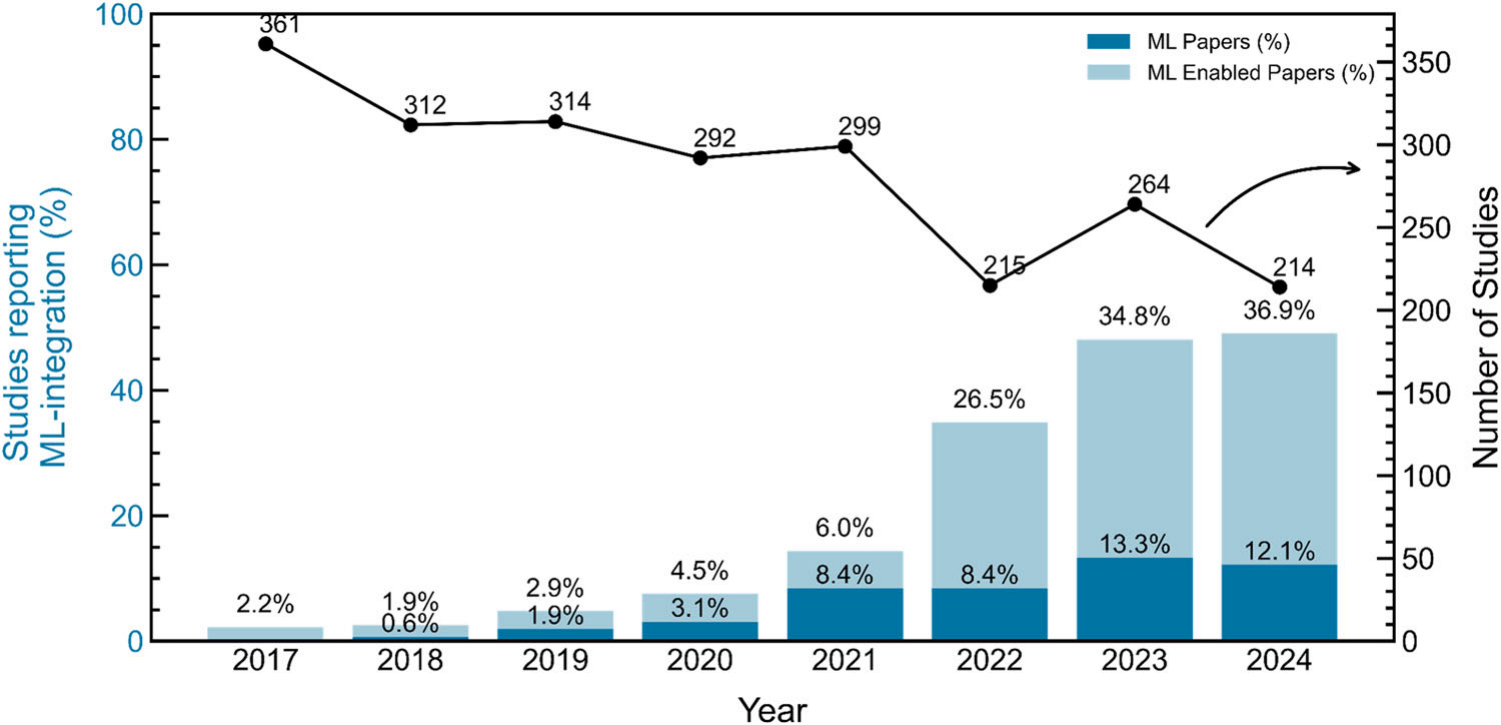
Stacked barplot showing the percentage of included studies each year that reported using machine learning (ML) or ML‐enabled tools for any evidence synthesis task. The dark blue portion of each bar represents studies that explicitly reported using ML, either through ML‐embedded tools or by enabling ML functionality in ML‐enabled tools. The light blue portion represents studies that reported using ML‐enabled tools without specifying whether ML features were used. The scatter plot (right *y*‐axis) shows the total number of included studies published each year.

The scatter plot in Figure 1 shows the number of included publications by year. A decreasing trend in the number of included publications per year is observed over the study period. This pattern is partially attributable to our inclusion criteria, which permitted the original version of a review to be included while excluding subsequent updates. For example, in 2017, 55 original reviews were included, whereas their later updates were excluded. This approach was used to ensure that methodological changes in the review process were accounted for while preventing duplication of findings from the inclusion of multiple versions of the same review.

The barplot in Figure 1 shows the percentage of included studies each year that reported using ML tools or workflows for any evidence synthesis task (raw data are provided in Table 1). The dark blue sections of each bar represent studies that explicitly reported using ML—either by employing ML‐embedded tools (in which ML is integral and cannot be disabled) or by enabling ML features in ML‐enabled tools. The light blue sections represent studies that reported using ML‐enabled tools but did not specify whether ML functionalities were actively used. A notable increase in ML‐enabled tools is observed between 2021 and 2022, rising from 6% to 26% of included papers. This shift is largely attributed to Covidence [82], one of the most widely reported screening tools, which introduced ML functionalities for Randomized Control Trial (RCT) classification [77] and prioritized screening [76] in 2022. Before this update, we classified Covidence as an “automated” tool (i.e., incorporating rule‐based automation but not ML); following the update, it was reclassified as ML‐enabled (indicating that optional ML features were integrated into the software). Similarly, we found documentation indicating that Rayyan introduced active‐learning‐based prioritized screening by the year 2020 [83, 84, 85], thus studies reporting usage of Rayyan before 2020 were classified as “automated” and “ML‐enabled” after.

**Table 1:** Summary of included studies by publication year, including total number of studies, number and percentage of studies reporting the integration of machine learning (ML), and number and percentage of studies reporting the use of ML‐enabled tools without specifying whether ML functionalities were used. Percentages are calculated relative to the total number of studies published in each year.

Year	Total papers	Studies reporting ML usage		Studies reporting ML‐enabled tool usage	
		Number	Percentage	Number	Percentage
2017	361	0	0	8	2.2
2018	312	2	0.6	6	1.9
2019	314	6	1.9	9	2.9
2020	292	9	3.1	13	4.5
2021	299	25	8.4	18	6.0
2022	215	18	8.4	57	26.5
2023	264	35	13.3	92	34.8
2024	214	26	12.1	79	36.9

Due to the inherent publication lag in research, some studies classified as ML‐enabled likely conducted their methodology before the relevant ML features were introduced. To ensure consistency, we classified tools based on the year ML functionalities were officially released. To estimate the potential misclassification error arising from this lag, we allowed a 2‑year “buffer” after each tool's ML release. For Rayyan, in the 2 years following its 2020 rollout, 15 of 32 ML‑enabled classifications (4 in 2020 and 11 in 2021 out of a total of 13 and 18 studies) may have preceded the feature's availability—an upper bound error of ~48%. Likewise, for Covidence, in the 2 years following its 2022 rollout, 107 of 149 ML‑enabled classifications (30 in 2022 and 77 in 2023 out of 57 and 92 studies) may reflect studies conducted before its ML update—an upper bound error of ~72%. In practice, real‑world misclassification is likely lower as some studies would align with the tool's released features; nonetheless, this 2‑year window provides an estimate of uncertainty in our reclassification approach.

For all studies reporting the use of ML‐enabled tools without explicitly mentioning use of ML functionalities, it remains unclear whether ML features were actively used but not reported, or simply not used at all. This limitation is further discussed in Section 3.4.2.

**Figure 2:**
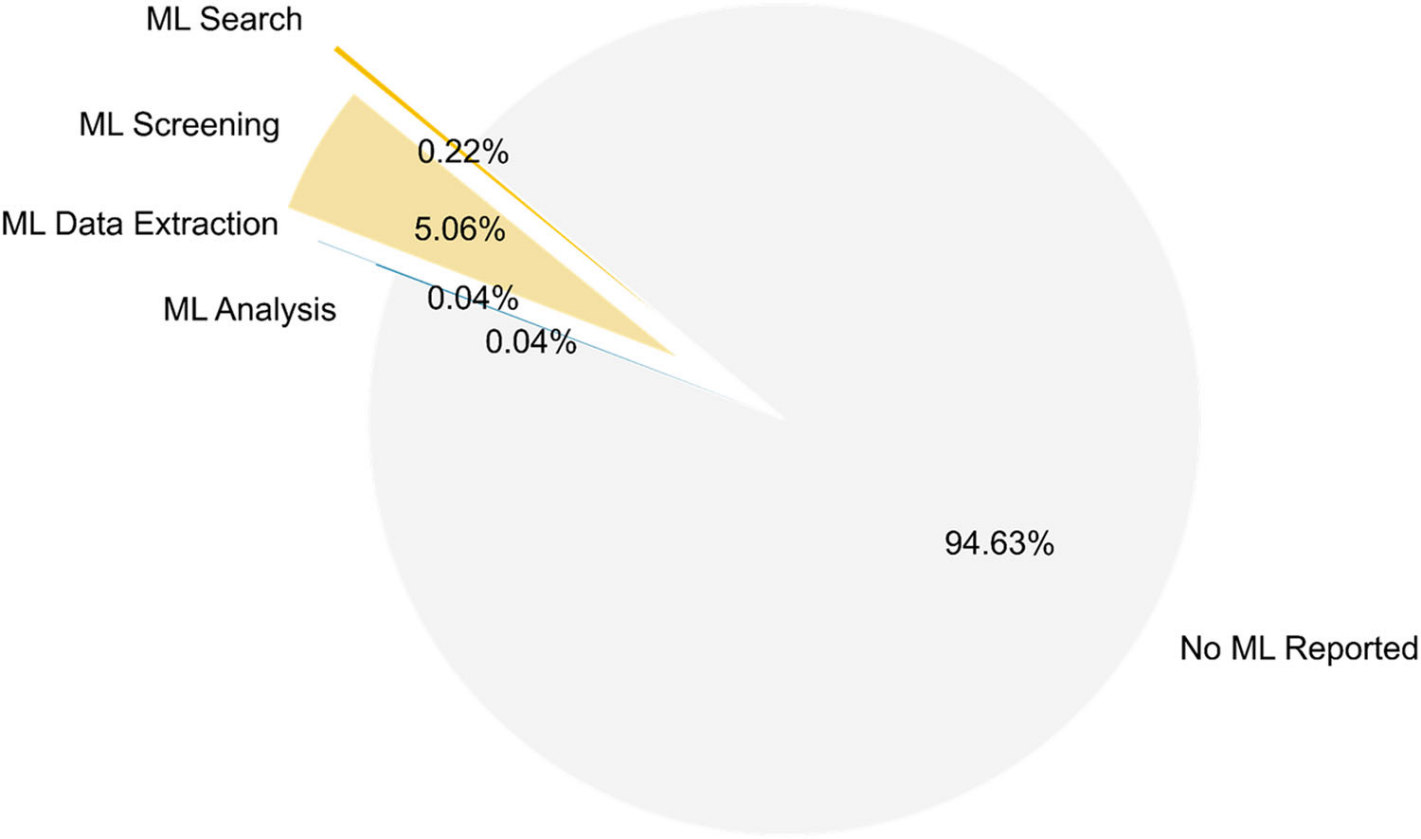
Pie chart showing the percentage of studies that explicitly reported the integration of machine learning (ML) during search, screening, data extraction, and analysis tasks relative to those that report no ML integration.

Of the 2271 studies analyzed, ML was explicitly reported in ~5% (121 studies). Among these, 115 studies (~95%) utilized ML during screening, five for search, and one each for data extraction and analysis. Only two of these studies [86, 87] reported ML usage across multiple steps (search and screening). The pie chart in Figure 2 shows this distribution, where ~95% of studies did not explicitly report the use of ML for any evidence synthesis task.

**Figure 3:**
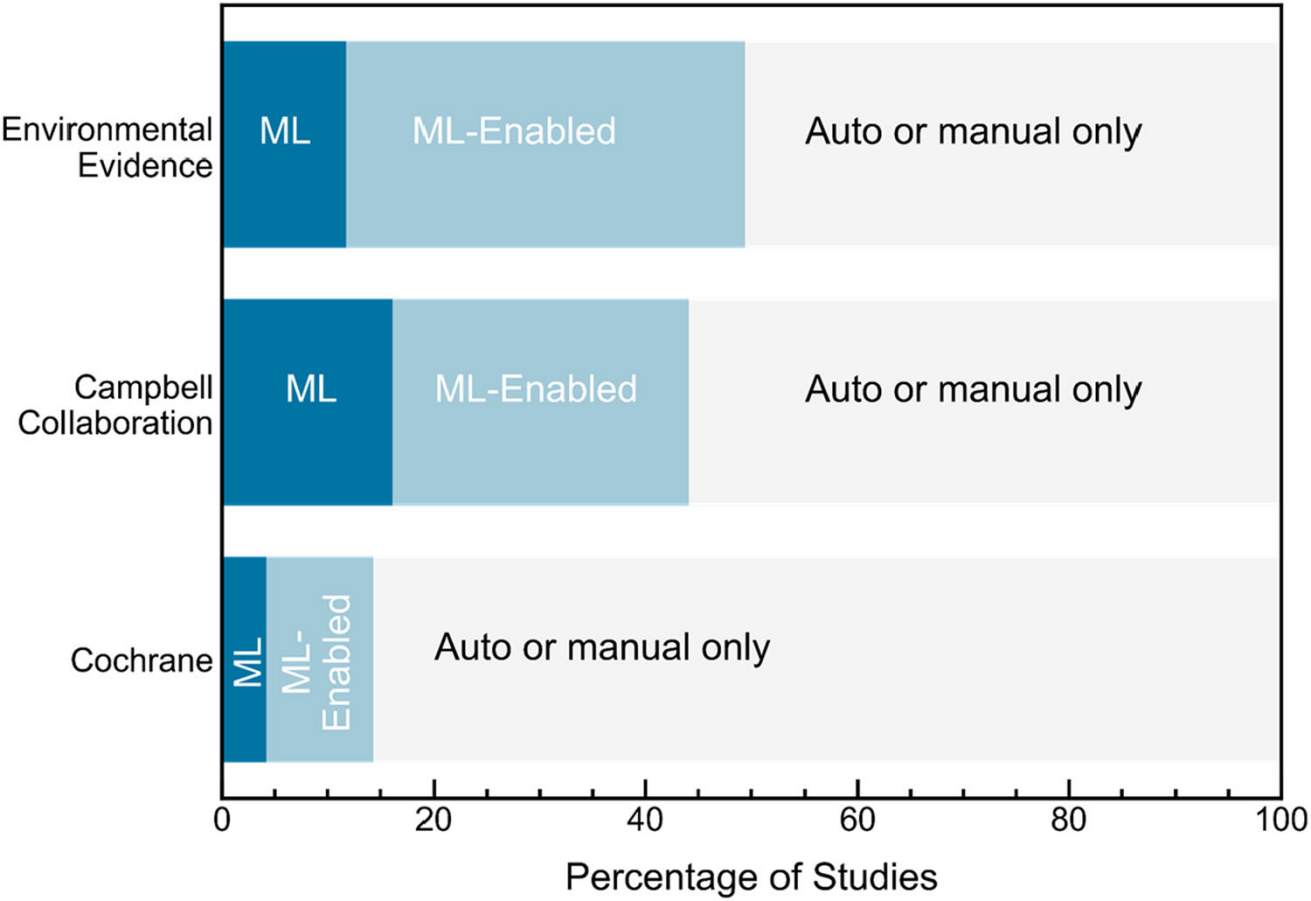
Barplot showing the percentage of studies from each source that reported using tools with varying levels of machine learning (ML) integration for any evidence synthesis task. Dark blue regions represent studies that explicitly reported ML use—either through ML‐embedded tools (where ML is integral and cannot be disabled) or by enabling ML features in ML‐enabled tools. Light blue regions represent studies that used ML‐enabled tools (tools with built‐in ML capabilities) but did not specify whether ML features were actively used. Light gray regions indicate studies that either used only rule‐based automation (non‐ML tools) or did not report using any tools (“auto or manual only”).

A barplot showing the reported use of ML by source is shown in Figure 3. As in Figure 1, the dark blue sections of each bar represent studies that explicitly reported using ML, whereas the light blue sections represent studies that used ML‐enabled tools but did not specify whether the ML features were actually used. Studies that relied exclusively on non‐ML automation or did not report any tool usage are shown in light gray. Overall, 121 studies (~5%) explicitly reported ML usage, while 282 studies (~12%) reported use of ML‐enabled tools without reporting whether ML functionalities were used. The reporting rates varied by source, where studies published in *Campbell Systematic Reviews* had the highest rate of explicit ML usage (~16%), followed by *Environmental Evidence* (~12%) and *Cochrane Database of Systematic Reviews* (~4%). The highest rate of ML‐enabled tool usage (without reported ML integration) was observed in *Environmental Evidence* (~38%), followed by *Campbell Systematic Reviews* (~28%) and *Cochrane Database of Systematic Reviews* (~10%).

### 3.3 Search Automation

Literature searches in evidence synthesis are intended to be a systematic, comprehensive, and transparent process designed to identify all relevant studies while minimizing bias [88, 89]. As it forms the foundation for study selection, this step is critical for ensuring methodological rigor [90]. An effective search strategy balances recall, which captures as many relevant studies as possible, and precision, which limits the number of irrelevant studies that must be screened [52].

**Figure 4:**
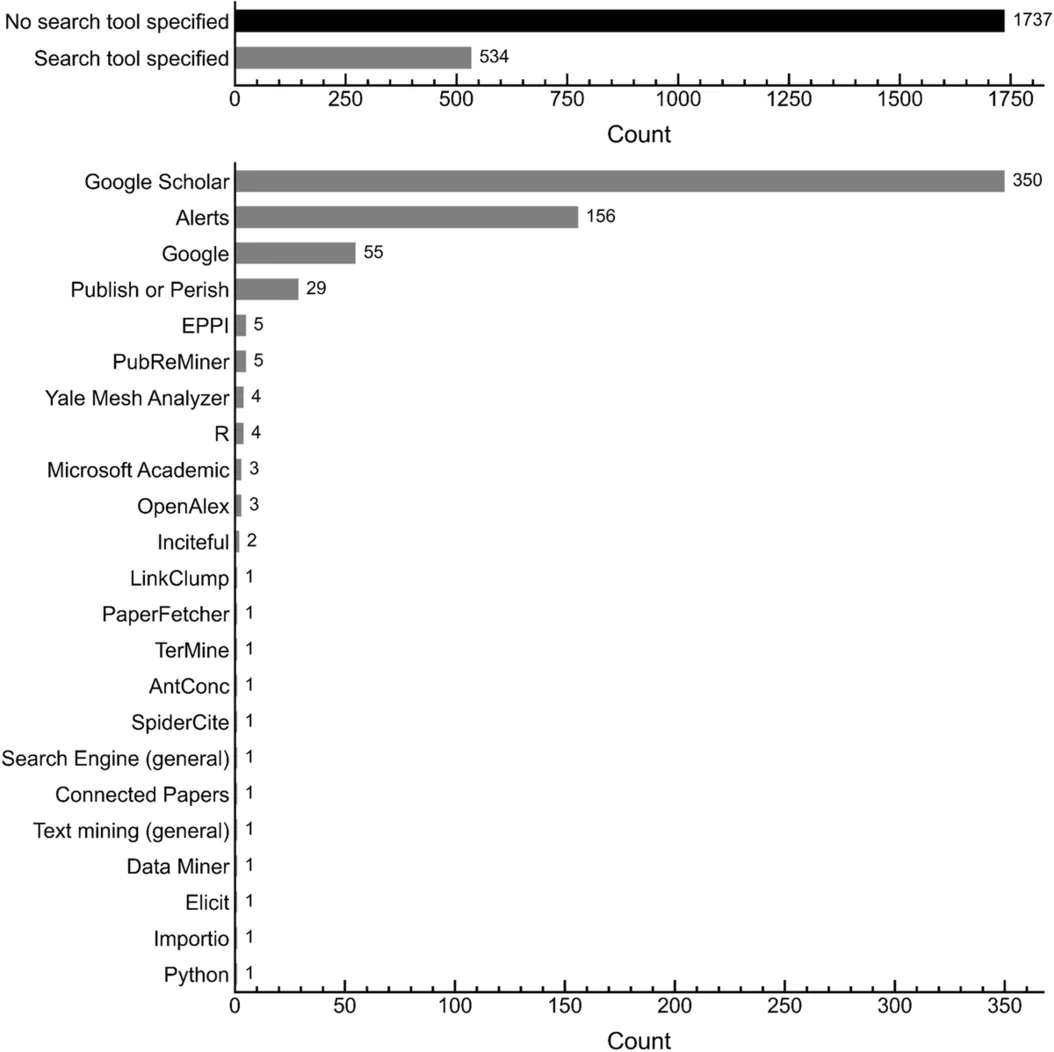
Bar plots showing reported tool usage for search. The top plot shows the number of studies that did (gray) and did not (black) report tool usage. The bottom plot shows the frequency of reported tool usage among studies that reported usage. Use of standard databases (e.g., Web of Science, Scopus) were not recorded.

Figure 4 shows bar plots of reported tool usage during the search phase. The top panel shows the number of studies that reported using search tools (gray) compared to those that did not (black). The lower panel shows the frequency of specific tools among studies that reported usage. Although most studies used standard bibliographic databases (e.g., Web of Science, Scopus), these were not recorded due to their widespread use and well‐established methodologies for searching and reporting [91]. Overall, ~31% of studies (*n* = 534) reported the use of search tools outside of standard bibliographic databases. The use of automated alerts and notifications were reported by 156 studies to help track new publications, while tools like Publish or Perish, Import.io, LinkClump, R packages (“greylitsearcher” [92] and “tidyverse/rvest” [93]) and use of Python scripts were reported by 34 studies for simplifying the bulk retrieval of search results. Remaining tools can be classified as search engines, text‐mining tools, and citation‐based discovery tools and are described in more detail in the following.

#### 3.3.1 Search Engines

Unlike traditional bibliographic databases, search engines such as Google and Google Scholar use proprietary ML algorithms to refine search results dynamically. These algorithms analyze citation networks, keyword relevance, document structure, and user interactions to adjust rankings over time [94, 95, 96]. Google Scholar, in particular, continuously updates its indexed content via web crawling rather than using a static database. This process enhances search coverage, particularly for gray literature [95, 96, 97], but also introduces challenges related to transparency and reproducibility of searches [94, 95, 96, 98, 99]. Because search engine results can vary over time or across different users, their use in systematic searches should be documented carefully [95].

As shown in Figure 4, Google and Google Scholar were among the most frequently reported search tools, with ~16% of studies reviewed reporting these tools. Among the 366 studies that reported using one or both of these tools, 58 studies used Google Scholar exclusively for citation searching, whereas 308 studies reported using Google or Google Scholar for literature retrieval via keyword searching. The level of documentation for studies using Google/Google Scholar for keyword searching varied considerably. Of these 308 studies, 197 (~64%) provided only minimal search details, such as search terms and/or the date searched. Twenty studies (~6%) explicitly labeled their approach as “non‐systematic,” acknowledging search reproducibility challenges. The remaining 91 studies (~30%) provided additional methodological details. Eighty‐six studies reported the number of search results or pages screened, five studies described using a stopping criterion based on the number of consecutive irrelevant results encountered, and nine studies employed private‐mode browsing to mitigate personalization biases. Three studies [100, 101, 102] referenced the recommendations by Haddaway et al. [95] who emphasized the importance of documenting exact search parameters, detailing the number of results or pages screened, noting search dates and terms, and specifying whether the standard or advanced search feature was used. The authors further suggest that gray literature often appears beyond the first 20–30 pages of results, so scanning at least 200–300 records may be necessary for a thorough search. These strategies collectively seek to maximize transparency and reproducibility when using search engines whose algorithms are neither fully disclosed nor static. One study noted that they did not use Google due to search reproducibility‐related concerns [103].

#### 3.3.2 Text‐Mining Tools

Text‐mining tools typically use Natural Language Processing (NLP) approaches to extract keywords and refine search strategies. For example, Yale MeSH Analyzer [104] is a non‐ML (or rules‐based) tool that helps optimize searches in medical databases by examining Medical Subject Headings (MeSH) terms, while TerMine and AntConc [105] (also rules‐based tools) perform corpus analysis (i.e., the systematic examination of text to identify patterns, frequencies, or contexts) and keyword extraction to enhance the specificity of systematic searches. PubReMiner [106] is another text‐mining resource that retrieves frequently used keywords, authors, and journals from PubMed abstracts, allowing researchers to refine and expand their search terms. A total of nine studies [107, 108, 109, 110, 111, 112, 113, 114, 115] reported the use of these non‐ML text‐mining tools for optimizing search queries. Elicit [116, 117] is an AI‐driven tool that summarizes key points of articles and suggests other relevant papers; one study reported use of this tool, indicating that it was an AI‐driven tool used to “search for references on the Internet” [118]; further details of how the tool was used or the underlying algorithms the tool leverages were not provided.

#### 3.3.3 Citation‐Based Discovery Tools

Citation‐based discovery tools rely on citation networks for identifying related studies, often through forward or backward citation searching. Approximately 83% of studies (*n* = 1894) either did not report tool usage or otherwise indicated that they manually searched reference lists, while ~8% (*n* = 182 studies) did not report conducting citation searching. Of the 195 studies that reported using a tool for citation searching, 125 (~64%) studies reported using a standard database (e.g., Scopus, Web of Science) and 84 (~43%) studies reported using Google Scholar.

Five studies reported using EPPI‐Reviewer for citation searching [86, 87, 119, 120, 121], though one study did not report further details of how the tool was used [120]. Three studies reported using the EPPI‐OpenAlex integration [86, 87, 121], which leverages OpenAlex [122], an open bibliometric database, to enhance citation searching. This integration applies ML algorithms to predict the relevance of papers within a citation network, suggesting articles with high relevance rankings [122, 123, 124]. However, only two of the three studies explicitly mentioned the ML functionality of the tool [86, 87]. Additionally, one study [119] reported using an EPPI‐Reviewer integration with Microsoft Academic, which applies ML in a manner similar to that of Open Alex [123], but did not specify that the tool utilized ML. Another study reported using a Microsoft Academic integration through The Human Behaviour Change Project [125], which employs NLP and automated feature extraction to identify relevant studies from citation networks [126].

Two studies [100, 101] reported using Inciteful [127, 128] a graph‐based citation search tool that incorporates ML, specifically ML‐driven link prediction algorithms and network‐based similarity metrics. While both studies acknowledged using an ML‐based tool for screening, neither explicitly discussed the ML functionality embedded within Inciteful.

Four studies reported the use of non‐ML automation tools for citation searching. Three studies [129, 130, 131] used tools that support automated snowballing for forward and backward citation searches, including: the R package, “citationchaser” [132], SpiderCite from SR Accelerator [133], and Paperfetcher [134], and one study reported using Connected Papers [135]. Connected Papers [136] generates force‐directed graphs to illustrate relationships between papers based on bibliographic coupling (measuring how often papers cite the same references) and co‐citation analysis (measuring how often papers are cited together). While the study described Connected Papers as ML‐driven, the tool's documentation describes a reliance on non‐machine‐learning graph‐based algorithms to analyze relationships between papers [136, 137].

#### 3.3.4 Reference Management and Deduplication

The use of reference management tools was reported in ~39% of included studies. Here, we defined reference management software as any digital tool designed to store, organize, and manage bibliography references throughout the review process. These tools fall into two categories: (1) standalone reference management software primarily used for organizing citations (e.g., EndNote, Zotero) and (2) systematic review platforms with built‐in reference management functionalities alongside screening, data extraction, and other review tasks (e.g., Covidence, EPPI‐Reviewer). The most commonly reported software used for reference management was Covidence, followed by EndNote and EPPI‐Reviewer.

Approximately 17% of studies reported using an automated deduplication process, though usage varied by source: ~14% of *Cochrane Database of Systematic Reviews*, ~40% of *Campbell Systematic Reviews*, and ~65% of *Environmental Evidence* reviews reported utilizing automation for deduplication.

### 3.4 Screening Automation

The screening process in evidence synthesis involves selecting studies for inclusion based on predefined eligibility criteria [138]. As described earlier, this process is often conducted in two stages. First, screeners evaluate studies by reviewing their titles and abstracts. Studies that pass this initial screening then undergo a full‐text review to confirm eligibility [138, 139]. To ensure consistency and minimize bias and errors, screening is often conducted by two or more independent reviewers who perform duplicate screening for at least a subset of studies [140, 141, 142, 143].

**Figure 5:**
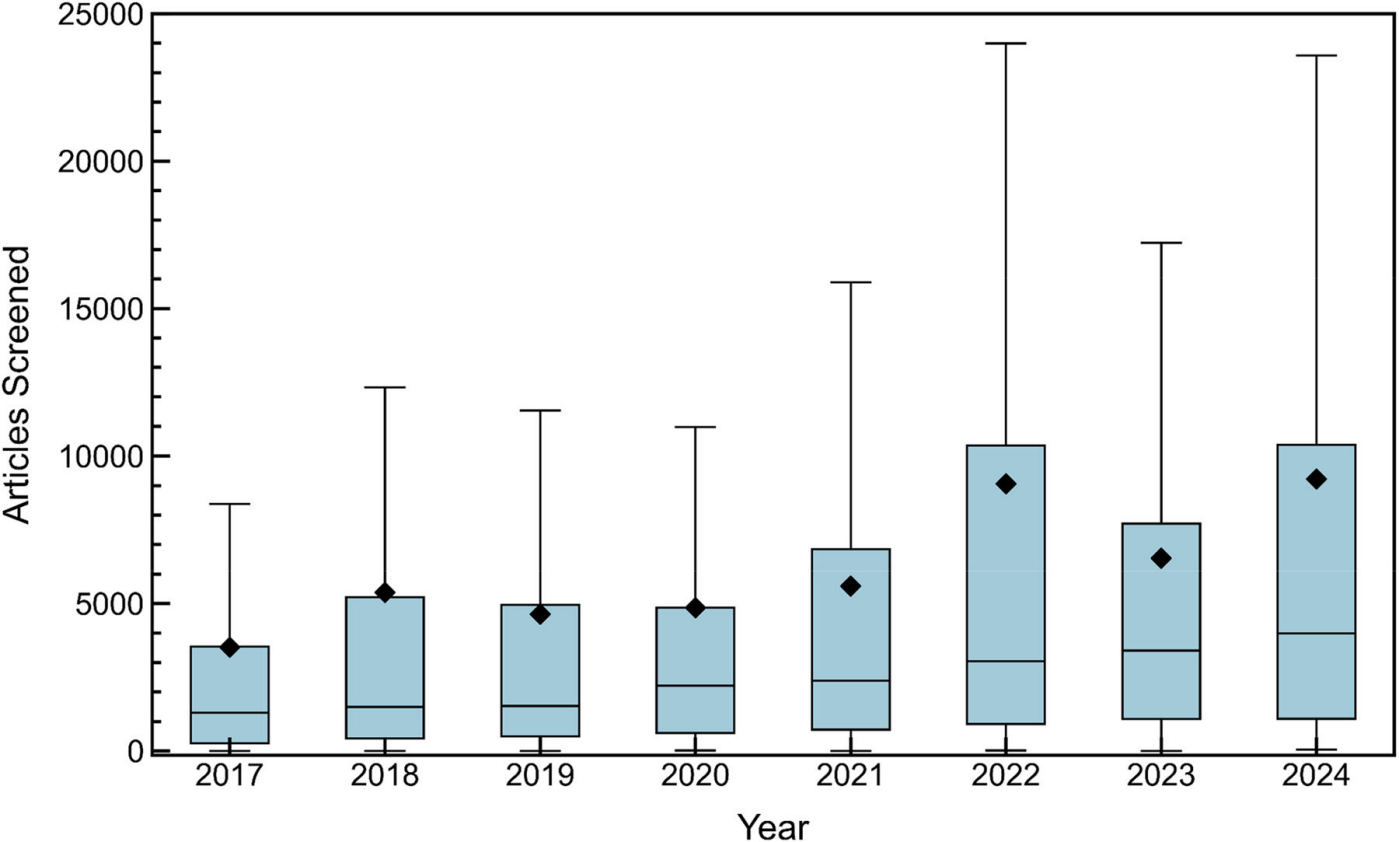
Box‐and‐whisker plot showing the distribution of the number of search results after duplicate removal (representing number of records screened, by humans or otherwise) per study across publication years. The boxes represent the interquartile range (IQR), which spans from the first quartile (Q1, 25th percentile) to the third quartile (Q3, 75th percentile), and the horizontal lines inside the boxes denote the medians. The whiskers extend to the minimum and maximum values within 1.5 times the IQR beyond Q1 and Q3. The mean markers (black diamonds) represent the average number of articles screened per year. Outliers (data points beyond 1.5 × IQR from Q1 and Q3) are not shown for clarity.

Figure 5 shows a box‐and‐whisker plot that illustrates the distribution of search results after duplicate removal (i.e., the number of records screened, whether by humans or automated tools) per study across publication years. The boxes represent the interquartile range (IQR), which spans from the first quartile (Q1, 25th percentile) to the third quartile (Q3, 75th percentile), the horizontal lines inside the boxes denote the medians, and black diamonds show mean values. Whiskers extend to the minimum and maximum values within 1.5 times the IQR beyond Q1 and Q3; outliers (data points falling outside these boundaries) are omitted for clarity. A general upward trend is observed for both mean and median values, suggesting that the number of articles screened per study has increased over time. Additionally, the upper‐bound whiskers have lengthened in recent years, suggesting a growing variance in screening demands. Across all years, studies screened an average of 5772 ± 11,154 articles. In 2017, the average number of articles screened per study was 3,514 ± 7234, increasing to 9377 ± 16,366 in 2024. Using an estimated title/abstract screening time of 30 s per article per reviewer [144], the average title/abstract screening time per study was 29 ± 60 h in 2017, increasing to 78 ± 136 h in 2024—an increase of ~169% over 7 years. When two independent reviewers are used—as is most often reported—the total time required for title/abstract screening roughly doubles. These findings reflect increasing screening demands over the study years, likely driven by the growing volume of scientific literature [25, 26]. Around 2019, Cochrane introduced Randomized Control Trial (RCT) search filters to increase the search precision; these have acted to streamline screening efforts [145], possibly moderating these trends.

#### 3.4.1 Screening Tool Usage

**Figure 6:**
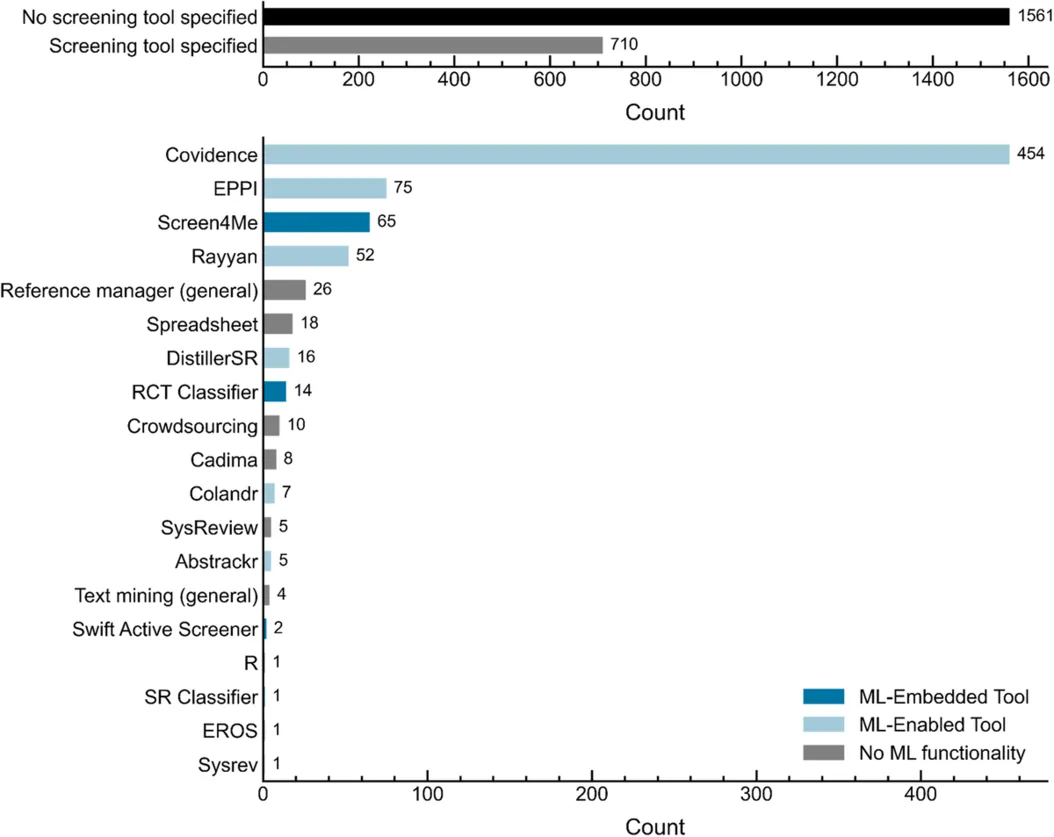
Bar plots showing reported tool usage for screening. The top plot shows the number of studies that did (gray) and did not (black) report tool usage. The bottom plot shows the frequency of reported tool usage among studies that reported screening tool usage. The bar colors indicate whether the tool is designed specifically to use machine learning (“ML‐embedded tool,” dark blue), includes optional machine learning functionality (“ML‐enabled,” light blue), or has no machine learning functionality (gray).

Screening tools are designed to support the selection of studies during title/abstract and full‐text screening. These tools facilitate the application of eligibility criteria by allowing reviewers to assess study relevance, record inclusion/exclusion decisions, resolve conflicts, and monitor screening progress. The bar plots in Figure 6 show reported screening tool usage. The top panel shows the number of studies that reported using screening tools (gray) compared to those that did not (black), while the lower panel shows the frequency of specific tools among studies that reported usage. Overall, only about 31% of studies reported using a tool for screening. However, the proportion varied by source: approximately 27% of *Cochrane Database of Systematic Reviews*, ~61% of *Campbell Systematic Reviews*, and ~68% of *Environmental Evidence* reviews reported using screening tools.

The use of screening tools has evolved over time; researchers began using spreadsheets in the late 1980s, followed by citation management tools in the early 2000s (e.g., Zotero [146], Endnote [147]), and specialized screening or systematic review software in more recent years [27, 138]. The data set captures tool usage across this spectrum. Of the 710 studies that reported using a tool, 12 (~2%) used only a spreadsheet, while 22 (~3%) relied solely on a citation manager. By source, the proportion of studies that used only spreadsheets or citation managers was ~3% for Cochrane Database of Systematic Reviews, ~9% for Campbell Systematic Reviews, and ~14% for Environmental Evidence reviews.

A growing number of specialized screening software tools now integrate advanced features such as automatic assignment of records to reviewers to facilitate duplicate review, conflict resolution, automatic inter‐rater reliability calculations, and progress tracking [51, 138, 148]. These tools enhance efficiency and improve the transparency and reproducibility of the screening process [148]. Of the 710 studies that reported using a screening tool, 676 (~95%) used at least one specialized tool. Covidence [82] was the most frequently reported tool, used in ~67% of studies, followed by EPPI‐Reviewer [149] (~11%) and Rayyan [83] (~7%).

The distribution of tool usage varied by source: Cochrane reviews most frequently reported using Covidence (~78%), followed by Rayyan (~7%); Campbell Collaboration reviews most frequently reported EPPI‐Reviewer (~44%), followed by Covidence (~25%); and Environmental Evidence reviews most frequently reported EPPI‐Reviewer (~32%), followed by Rayyan (~17%). Other reported software tools, in decreasing order of frequency, included: DistillerSR [150], Cadima [151], Colandr [152], SysReview [153], Abstrackr [154], Swift Active Screener [80], and Sysrev [73]. These tools are described further in the following section.

#### 3.4.2 ML‐Assisted Screening

The bar colors in Figure 6 reflect the classification of the screening tools themselves, based on their level of ML integration—not on how individual studies described their use. Dark blue represents tools classified as ML‐embedded, meaning ML is core to the tool's functionality and cannot be disabled. Light blue represents ML‐enabled tools, which have built‐in ML capabilities that can be turned on or off by the user. Tools without ML functionality (those that rely on rule‐based automation or manual processes) are shown in gray. As previously noted, ~95% of studies that explicitly reported using ML applied it during the screening phase. Among these, 82 (~71%) reported using ML‐embedded tools.

The most frequently reported ML‐embedded tool was Cochrane's Screen4Me workflow [78, 79], which integrates ML and crowdsourcing to identify randomized controlled trials (RCTs). The workflow employs an RCT classifier, an ML model based on SVMs and trained on a large data set of previously categorized studies, to distinguish RCTs from non‐RCTs by analyzing the text of study abstracts. This classifier assigns a score to each study; those exceeding a specified threshold are classified as “Possible RCTs” and proceed to further screening, while those below the threshold are automatically excluded [63, 72]. In total, 65 studies reported using the Screen4Me workflow, while an additional 14 studies used only the RCT classifier. Both Covidence and EPPI‐Reviewer integrate the RCT classifier, while EPPI‐Reviewer also includes a classifier for identifying systematic reviews [155], which functions similarly. One study reported using the systematic review classifier alongside the RCT classifier within EPPI‐Reviewer [156]. Lastly, two studies [100, 101] reported using Swift Active Screener, an ML‐embedded tool that employs active learning‐based prioritized screening that cannot be disabled [80]. Notably, all studies that reported using ML‐embedded tools explicitly acknowledged their use of ML.

Most of the specialized screening tools that were reported integrate optional ML functionality that users can disable. Typically, this takes the form of active learning‐based prioritized screening, where records are sorted by relevance to expedite the review process. Some tools enable ML by default, requiring users to actively turn it off. For example, Covidence introduced priority screening in 2022 [76, 77], automatically ranking records by relevance. If users proceed with the default ranking, they are leveraging ML functionality within the software. Other tools that incorporate optional ML functionalities include Abstrackr [55, 154, 157, 158], Colandr [152, 159], DistillerSR [56], EPPI‐Reviewer [160, 161], Rayyan [83], and Sysrev [73]. The level of transparency regarding ML usage—whether ML functionalities are enabled by default and how clearly users are made aware of their use—varies across these tools and has evolved over different software versions.

Among the studies that did not explicitly report integration of ML, 318 reported using ML‐enabled tools, and only 33 (~10%) reported utilizing ML functionalities. As discussed in Section 3.2, tools that introduced ML capabilities during the study period were initially categorized as non‐ML but were reclassified as ML‐enabled from the year these functionalities were introduced. Given the publication lag in research, some studies classified as using ML‐enabled tools likely conducted their methodology before these tools incorporated ML functionality. As a result, the 10% estimate of studies reporting use of ML functionalities within ML‐enabled tools is likely an underestimate. Nonetheless, this disparity is substantial. Only one study explicitly stated that it did not use ML during the review process, and this study did not report using any ML‐enabled tools [162].

#### 3.4.3 Duplicate Screening in ML‐Assisted Screening

**Figure 7:**
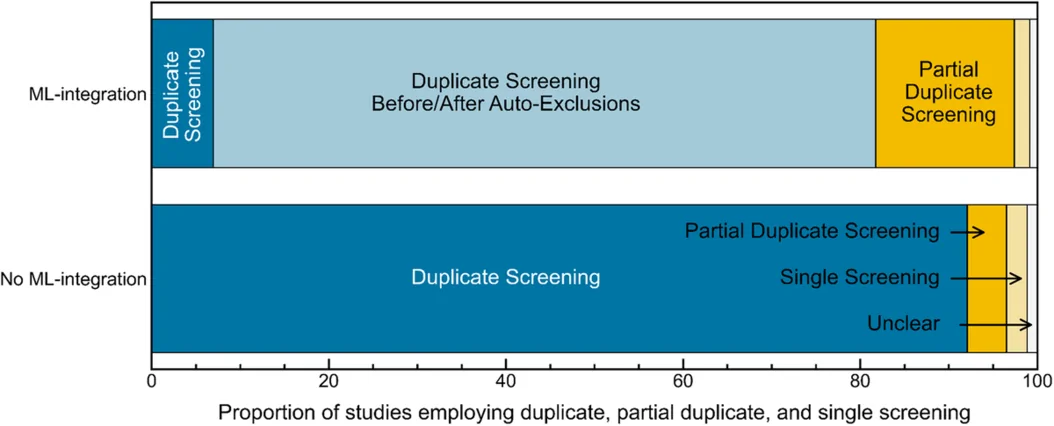
Comparison of screening approaches used in studies reporting ML‐assisted versus non‐ML‐assisted screening. Bars represent the proportion of studies that employed duplicate screening (dark blue and light blue), partial or selective duplicate screening (dark yellow), or single screening (yellow) during the title/abstract and full‐text screening stages, and white regions indicate the proportion of studies for which screening details were unclear.

Figure 7 shows a comparison of screening methods used in non‐ML‐assisted versus ML‐assisted screening. Among the 2156 studies that conducted screening without ML assistance, ~92% employed duplicate screening throughout both the title/abstract and full‐text screening stages (dark blue portion of the non‐ML assisted bar in Figure 7), ~7% did not use duplicate screening (yellow portion of the bar in Figure 7), while the screening approach was unclear in the remaining ~1% of studies (white portion of the bar in Figure 7). The extent of duplicate screening varied across review types, with systematic reviews reporting the highest levels of duplicate screening at ~94%, followed by rapid reviews at ~72% and evidence and gap maps at ~37%. Differences were also observed across sources, with ~96% of *Cochrane Database of Systematic Reviews*, ~80% of *Campbell Systematic Reviews*, and ~16% of *Environmental Evidence* reviews employing duplicate screening. The lower prevalence of duplicate screening in *Environmental Evidence* reviews is likely driven by the fact that ~66% of these reviews were evidence and gap maps, which often require screening of a larger volume of studies compared to systematic and rapid reviews.

**Figure 8:**
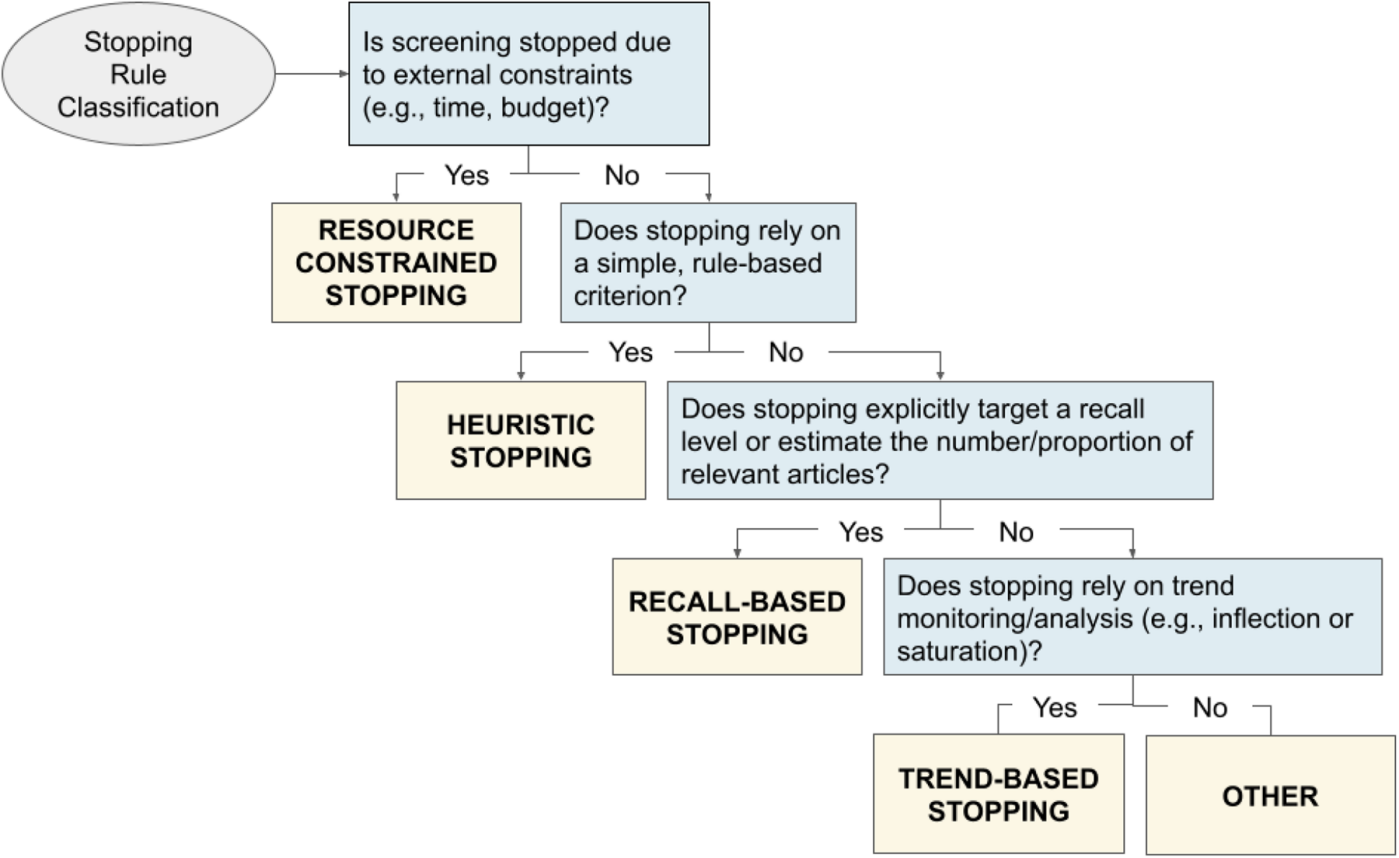
Decision tree for classification of ML‐assisted screening studies by their stopping rules. Each blue rectangle on the right represents a decision question and each yellow rectangle on the left represents the final classification.

Overall, 115 studies reported using ML‐assisted screening, including 82 Cochrane, 24 Campbell, and 9 Environmental Evidence reviews. ML‐assisted screening methods varied in their approach to study selection, with some studies using ML to prioritize and rank records for screening while still manually screening all records and others auto‐excluding records based on their predicted likelihood of inclusion. Nineteen (~17%) studies that used ML‐assisted screening reviewed all records manually, using ML to prioritize the review of records with the highest likelihood of inclusion [87, 119, 120, 163, 164, 165, 166, 167, 168, 169, 170, 171, 172, 173, 174, 175, 176, 177, 178]. One of these studies reported using DistillerSR's “Check for Screening Errors” function [150] to ensure that relevant studies had not been mistakenly excluded by human screeners [171]. Within this subset, seven studies applied duplicate screening throughout the entire process [119, 120, 169, 174, 175, 177, 178] (dark blue portion of the ML‐assisted bar in Figure 7), while the remaining 12 studies used duplicate screening for records of high predicted relevance and single screening for lower relevance records [87, 163, 164, 165, 166, 167, 168, 170, 171, 172, 173, 176] (dark yellow portion of the bar in Figure 7).

The remaining 96 studies, constituting ~83% of ML‐assisted screenings, applied ML to automatically exclude a portion of records, thereby reducing the total number of records requiring human screening. Among these, ~89% employed duplicate screening outside of auto‐exclusions (light blue portion of the ML‐assisted bar in Figure 7), with the remaining employing partial duplicate or single screening outside of auto‐exclusions (yellow portions of the bar in Figure 7).

#### 3.4.4 Stopping Criteria Used in ML‐Assisted Screening

A key consideration in the use of ML‐assisted auto‐exclusion is the definition and application of stopping criteria, which determine the conditions under which records are automatically excluded without human review. Of the 96 syntheses that auto‐excluded studies, 57 (~60%) did not specify the stopping criteria they applied. This lack of reporting undermines reproducibility and precludes any evaluation of auto‑exclusion performance. Approximately 70% and ~35% of Cochrane and Campbell Collaboration reviews, respectively, did not specify the stopping criteria used, whereas all Environmental Evidence reviews that auto‐excluded records specified the stopping criteria they followed. Moreover, only seven of the 96 studies (~7%) that auto‐excluded results addressed potential biases or limitations for doing so [100, 101, 179, 180, 181, 182, 183]. Ideally, stopping criteria should be chosen such that screening performance is optimized while the number of articles requiring manual review is minimized [53]. However, since stopping occurs before the full data set is reviewed, there is always a risk that relevant studies may be overlooked [184]. In general, a benchmark for ML‐assisted screening is to maintain recall rates of at least 95% to ensure that the process performs at a level comparable to human reviewers [53, 185].

We classified stopping criteria for those studies that described it (*n *= 38) based on the decision tree shown in Figure 8. These criteria broadly fell into four categories: (i) resource‐constrained stopping, (ii) heuristic stopping rules, (iii) recall‐based stopping rules, and (iv) trend‐based stopping. Resource‐constrained stopping (i) involves terminating screening due to external limitations such as time or budget constraints [186, 187]. Only one study described using this approach, where the decision to stop screening was dictated by time spent screening [188]. While resource‐constrained stopping is sometimes practically necessary, it increases the risk of excluding relevant studies since the decision to stop is based on practical constraints rather than an evaluation of whether sufficient relevant records have been identified [186, 187].

Heuristic stopping rules were commonly reported, with 13 studies (~34%) of those that described stopping criteria, following this approach. These stopping rules rely on predefined, simple decision criteria to determine when to stop screening [53, 144]. Heuristic stopping rules can be further classified as fixed (static) or data‐driven (adaptive). Fixed heuristic stopping rules are set before screening begins and do not adjust based on data set characteristics or model performance [144]. An example of a fixed heuristic rule is the exclusion of all studies below a specified probability threshold for inclusion. These probability‐driven thresholds introduce the risk of lower recall, as relevant studies that happen to fall below the predetermined cutoff may be excluded. Among the studies that used heuristic stopping rules, nine employed a fixed approach, with probability thresholds for auto‐exclusion ranging from below 0.1% to below 70% [180, 181, 183, 189, 190, 191, 192, 193, 194]. However, none of these studies provided empirical justification for their chosen cut‐off values, making their recall performance unknowable. The remaining four studies [110, 179, 195, 196] used a data‐driven heuristic approach, where stopping criteria were adjusted dynamically based on observed screening trends [53, 155, 197, 198]. A common example of data‐driven heuristics is discontinuing screening after a set number of consecutive irrelevant results, which assumes that once a saturation point is reached, the probability of encountering additional relevant records is low [197].

Recall‐based stopping rules were the most commonly reported approach, applied in 20 studies. These methods aim to discontinue screening once an estimated proportion of relevant abstracts has been identified. Within this category, 95% of studies used recall‐based statistical stopping [100, 101, 129, 182, 199, 200, 201, 202, 203, 204, 205, 206, 207, 208, 209, 210, 211, 212, 213], while only one study used a target recall threshold approach [214]. Recall‐based statistical stopping applies statistical tests to determine whether a predefined recall threshold has been met [80, 186]. Classifiers using this approach are calibrated to retain records until a specified percentage of relevant studies, as estimated by the model, have been identified. This method is designed to minimize false negatives, making it a more conservative approach than fixed probability thresholds. The probability score that determines when to stop screening varies based on model training and the cutoff required to achieve the stated recall percentage. The target recall method stops screening once a predefined recall threshold has been met, without requiring statistical confidence [215]. The recall threshold for this method is often derived from external validation datasets, prior systematic reviews, or expert domain knowledge. Other common recall‐based stopping rules, such as prevalence estimation [53, 216] and sampling‐based stopping [186], were not identified in the studies analyzed.

Outside of resource‐constrained stopping, trend‐based stopping methods were the least commonly reported, with only four studies describing this approach [217, 218, 219, 220]. Trend‐based stopping determines when to stop screening based on observed patterns in the data rather than predefined thresholds or external constraints [53, 215]. All studies that described trend‐based stopping criteria utilized a subtype of trend‐based stopping known as saturation‐based stopping, where screening is terminated when the number of newly identified relevant studies approaches zero, indicating that further screening is unlikely to yield additional relevant records. Because saturation assumes a monotonic decline in relevant hits, it can miss late‑appearing clusters of relevant records, but none of the four studies assessed this risk [186].

#### 3.4.5 Motivation and Barriers for Automation in Screening

Among the studies that used ML‐assisted screening, ~77% did not specify why automation was implemented. Twenty‐three studies stated that they integrated ML functionality to improve efficiency and reduce workload [87, 110, 164, 168, 174, 175, 179, 181, 182, 183, 189, 190, 217, 218, 219, 220, 221, 222, 223, 224, 225, 226, 227], while seven studies additionally noted that retrieval of a large volume of search results motivated their choice [179, 182, 183, 217, 225, 226, 227]. Two studies reported using DistillerSR's “Check for Screening Errors” function [150] to ensure that relevant studies had not been mistakenly excluded [129, 171].

Several studies provided reasons for choosing not to use automated processes. One study that had intended to use Covidence for (non‐ML) data extraction found the process too time‐consuming and instead opted for a manually piloted Excel‐based extraction form [228]. Four studies planned to use Cochrane's Screen4Me workflow but ultimately decided against it because their search results did not exceed 500–1,000 records, making ML‐assisted screening unnecessary [229, 230, 231, 232]. One study reported deviating from its protocol after finding that the priority screening function in EPPI‐Reviewer did not produce their expected results, leading the review team to discontinue its use [163]. Finally, one study reported issues with the accessibility of articles for automated text processing, as certain documents were formatted as image‐based PDFs or used nonstandard text encoding, making them unreadable by common digital tools [233]. The authors noted that this challenge poses a risk for future data accessibility as the volume of published research continues to grow.

### 3.5 Data Extraction Automation

**Figure 9:**
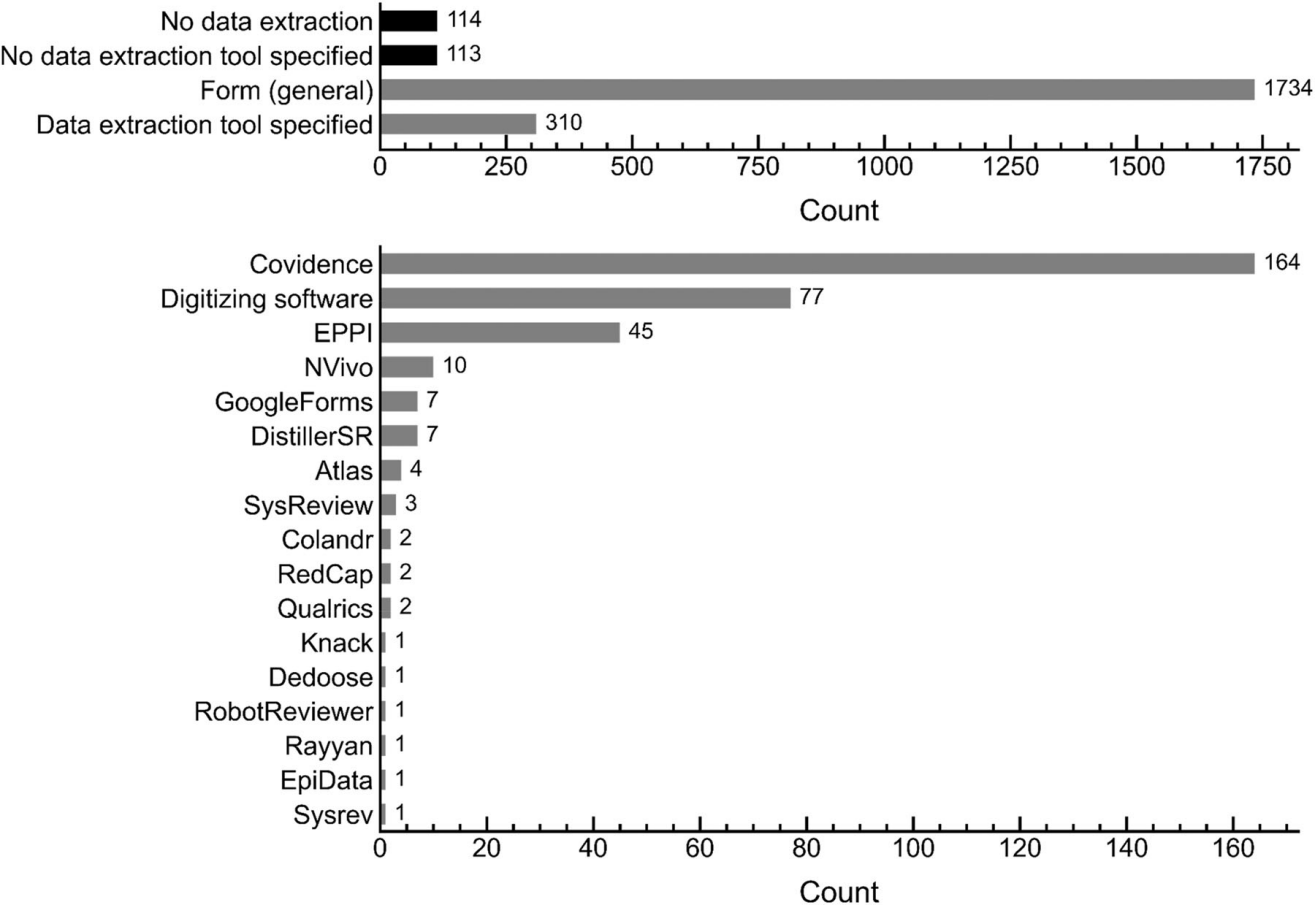
Bar plots showing reported tool usage for data extraction. The top plot shows the number of studies that: did not conduct data extraction (black), did not report tool usage for data extraction (black), reported using only a form for data extraction (gray), and the number of studies that reported tool usage beyond a general form (gray). The bottom plot shows the frequency of reported tool usage among studies that reported data extraction tool usage.

Data extraction in evidence synthesis involves systematically retrieving and organizing key information from included studies for later synthesis and analysis. Most studies analyzed (~76%) reported using only a standardized form for data extraction (sometimes forms were contained within a spreadsheet, though specific spreadsheet usage was not systematically tracked). Approximately 5% of studies did not conduct data extraction because they included no eligible studies, while another ~5% did not report any tool usage for this phase. Beyond standardized forms, 310 studies (~14%) reported using additional tools for data extraction (Figure 9, upper panel).

Only one study explicitly reported using ML for data extraction [234]. This study utilized RobotReviewer [235], an ML‐embedded tool designed to extract study characteristics and assess risk of bias in clinical trials. RobotReviewer applies natural language processing (NLP) to identify RCTs, extract participant data, interventions, and outcomes, and evaluate bias using Cochrane's Risk of Bias tool [236]. In that work, RobotReviewer correctly excluded one non‐RCT but misclassified two studies as RCTs, leading to data extraction errors. Thus, the extracted data were not fully usable in the final review, and risk of bias assessments were conducted manually.

The lower panel of Figure 9 shows the distribution of reported tools (beyond standardized forms). Systematic review software was the most frequently cited category for data extraction, with 223 studies employing such tools, including Covidence [82] (164 studies), EPPI‐Reviewer [149] (45 studies), DistillerSR [150] (7 studies), and fewer instances of SysReview [153], Colandr [152], RobotReviewer [235], Rayyan [83], and SysRev [73]. Fourteen studies reported using electronic forms or survey‐based tools, including GoogleForms [237], REDCap [238], Qualtrics [239], Knack [240], and EpiData [241]. Seventy‐seven studies reported using digitization software to extract data from figures, with WebPlotDigitizer [242] accounting for ~49% of these cases. Lastly, qualitative coding and thematic synthesis software were used in 15 studies, most commonly NVivo [243] (10 studies), followed by Atlas.ti [244] and Dedoose [245].

### 3.6 Language Translation

**Figure 10:**
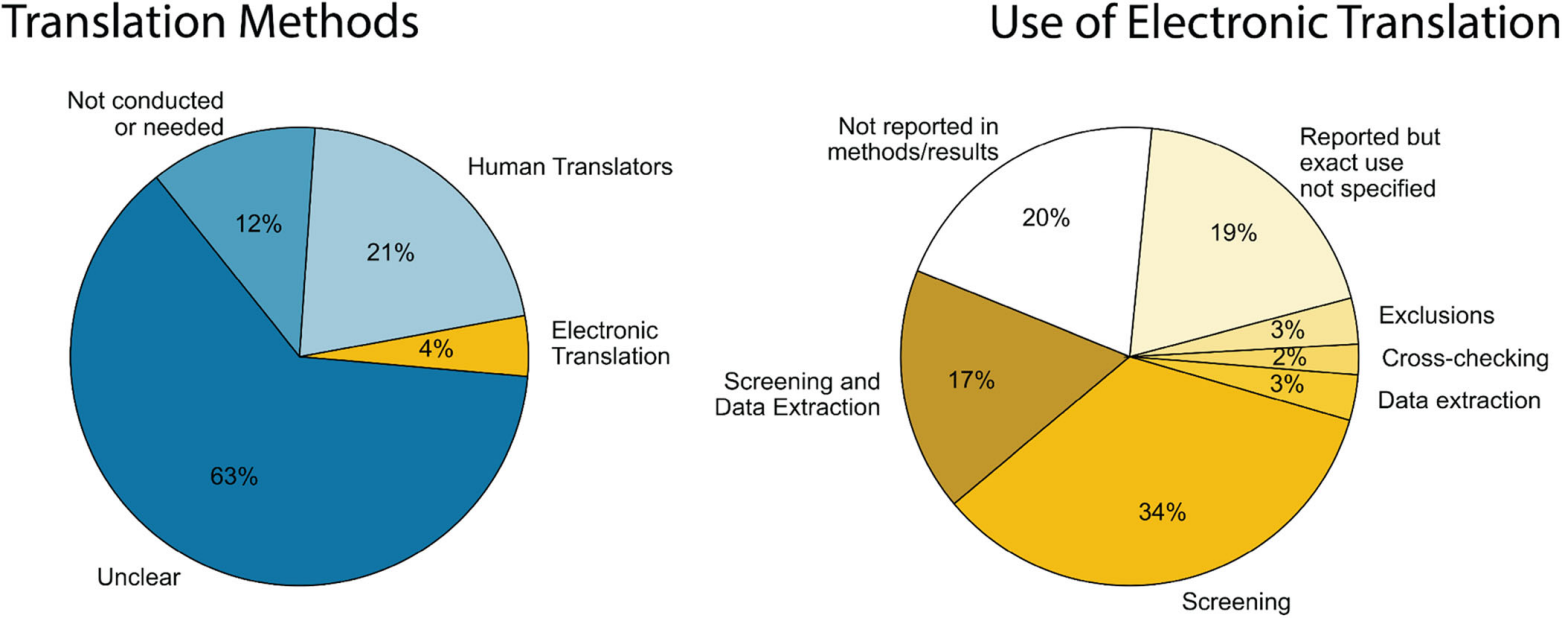
Pie charts showing (left) the distribution of translation and (right) electronic translation methods reported across included studies.

Language translation plays an important role in ensuring that non‐English studies are appropriately considered in evidence synthesis, minimizing language bias [246, 247, 248]. The pie chart on the left in Figure 10 shows the distribution of reported translation methods for included studies. Of the 2271 included studies, 584 (~25%) included non‐English language studies and performed translations, while ~12% of studies explicitly stated that they did not perform translations (either because of English‐language restrictions or because translations were not otherwise needed). In ~64% of studies, language restrictions were either not specified or not discussed, and the use of translation and associated methods was unclear. Approximately 21% of studies (*n* = 482) reported using human translators, though in ~28% of these cases, this information was only mentioned in acknowledgments or study‐level details in appendices rather than described within the methods sections. This lack of transparent reporting may obscure the potential influence of translation methods on study inclusion or interpretation, reinforcing the importance of clearly documenting translation procedures within methods sections.

Electronic translation tools were reported in 93 studies (~4%) either as the primary method or in combination with human translators. Google Translate [249] was the most frequently used tool, though DeepL [250], Baidu [251], and Yandex [252] were also reported. As shown in the pie chart on the right in Figure 10, ~20% of studies did not describe translation methods in the main text, mentioning them only in appendices or study‐level details. Another ~19% of studies described the use of electronic translation in methods or results sections, but did not specify whether it was applied for screening, data extraction, or both. Approximately 34% of studies used electronic translation exclusively for screening, relying on human translators when necessary for data extraction. Approximately 17% of studies used electronic translation for both screening and data extraction, while ~3% of studies applied it exclusively for data extraction.

A subset of studies employed electronic translation for specialized purposes. Specifically, ~2% of studies used electronic translation to cross‐check human translations, as they lacked the resources for two native speakers to validate each other's work, and ~3% of studies applied an English‐language restriction for study inclusion but used electronic translation to estimate the impact of potential language bias.

While electronic translation tools have improved access to non‐English content, their accuracy varies based on language, document complexity, and subject‐matter specificity [253, 254, 255]. This introduces potential risks for misinterpretation, particularly in data extraction or risk of bias assessments. Additionally, ML tools are often primarily trained on English‐language datasets, limiting their performance and reliability when applied to non‐English studies [37, 256, 257]. However, excluding studies due to an inability to translate them also contributes to language bias and undermines the comprehensiveness of evidence syntheses [247, 258]. Addressing the technical compatibility of tools with non‐English documents, the broader inclusion challenges posed by language limitations, and the need for transparent reporting of translation methods are all essential for promoting globally representative syntheses.

### 3.7 Synthesis and Analysis

The synthesis and analysis phase of the evidence synthesis process involves aggregating, interpreting, and summarizing findings from included studies to generate conclusions. This phase can include either or both qualitative and quantitative approaches, depending on the nature of the evidence and the research questions being addressed.

In this study, ~5% of included studies did not conduct any analysis because they did not identify any eligible studies and ~8% of studies did not report using a tool for analysis. The majority of studies (~87%) reported using at least one tool to support analysis and synthesis. Of these, ~94% reported using at least one tool for quantitative data analysis, and ~66% of studies reported using GRADEpro [259] to automate the generation of summary of findings tables based on manually extracted data. Only one study reported using ML in the synthesis/analysis phase of the evidence synthesis process [260]. ML was utilized through the use of EPPI‐Reviewer [149], which incorporates text mining and automated clustering functionalities to support synthesis tasks. These features were used to classify and group studies based on shared themes and keywords, facilitating the organization and interpretation of the evidence base.

### 3.8 Automation in Living Reviews, Updates, and Rapid Reviews

Living reviews and rapid reviews are two types of evidence synthesis that aim to provide up‐to‐date and timely summaries of research findings. Living reviews require continuous updates as new evidence becomes available, making automation useful for managing the ongoing workload [261, 262, 263]. Rapid reviews, conducted under time constraints, benefit from automation by reducing manual effort and accelerating the review process. Among the 26 living reviews included in this study, four reported integrating ML, all for ML‐assisted screening. Although this represents only ~15% of living reviews, the prevalence of ML use in living reviews is ~300% higher than in the overall sample, where only ~5% of studies employed ML techniques. Of eighteen rapid reviews included in this study, only one used ML; specifically, for data extraction [234].

Review updates are formal revisions of systematic reviews that incorporate new evidence. Unlike living reviews, which are continuously updated, these updates are published as new versions of the review. While review updates themselves were excluded from our primary analysis, we examined methodological changes in subsequent updates of included reviews to assess whether ML techniques had been newly integrated into the review process. A total of 126 review updates were identified in which the original review was published in 2017 or later, thus included in our analysis. To determine whether ML adoption had changed over time, we compared each original review to its most recent update, without examining intermediate versions. Among these, only five updates (~4%) incorporated ML techniques [264, 265, 266, 267, 268, 269, 270, 271, 272, 273], representing a slightly lower rate of ML integration compared to our overall study sample, where ~5% of studies integrated ML functionalities. Three original reviews published in 2017 integrated the Screen4Me workflow in their 2023 updates, incorporating ML‐assisted study identification for randomized controlled trials [266, 267, 268, 269, 270, 271]. Another review, originally published in 2019, implemented the Robot Search tool in its 2023 update to automatically remove studies that were unlikely to be RCTs [272, 273]. Lastly, a review first published in 2020 and updated in 2022 adopted a classification model to enhance efficiency in screening due to the rapidly increasing volume of COVID‐19 literature [264, 265].

## 4 Discussion

### 4.1 Summary of Findings

This study reviewed 2271 evidence syntheses published between 2017 and 2024 in three major sources—*Cochrane Database of Systematic Reviews*, *Campbell Systematic Reviews*, and *Environmental Evidence*—to assess the evolving use and reporting of automation and ML in evidence synthesis workflows. Our analysis focused on four main stages of evidence synthesis: search, screening, data extraction, and analysis/synthesis, with additional considerations given to translation tasks, reference management, and the integration of ML in living and updated reviews.

Despite growing interest in AI and ML, their adoption across evidence synthesis workflows remains limited, and inconsistently documented and reported. Only ~5% of studies explicitly reported using ML for any task, and most applications were confined to screening. The following sections address the study's three research questions, summarizing how automation and ML adoption has evolved over time (RQ‐1), which techniques and reporting practices are used across different review stages (RQ‐2), and what motivates or constrains researchers in adopting these technologies (RQ‐3).

#### 4.1.1 RQ 1. How Has the Adoption of Manual, Automated, and ML‐Based Techniques in Evidence Synthesis Evolved Across Key Review Stages (Search, Screening, Data Extraction, and Analysis)?

The average number of records screened per study increased by ~169% over the 7 year study period (2017‐2024). This increase likely contributed to greater adoption of ML‐assisted screening tools, such as Cochrane's Screen4Me workflow and ML‐enabled platforms like Covidence and EPPI‐Reviewer. The first explicit use of ML appeared in 2019 (~1% of studies that year), rising to ~12% by 2024, supporting our study timeframe of 2017–2024 to capture both early and more recent developments.

Screening was by far the most common evidence synthesis step for ML integration. Of the 121 studies that explicitly reported ML integration, 95% applied it during screening—primarily for prioritizing or auto‐excluding records. This aligns with previous methodological reviews showing that ML is most often applied at the screening stage [32, 46]. Approximately 31% of all included studies reported using any kind of screening tool (ML‐based or not). This is consistent with findings from Zhang and Nietzel [138], who found that only ~4% of evidence syntheses in educational research reported use of screening tools beyond basic citation managers, suggesting broader underreporting or limited uptake across disciplines.

Reported ML integration for carrying out search, data extraction, and synthesis tasks was minimal. Only five studies reported ML integration during search, and just one each reported its use for data extraction and synthesis. This limited uptake is consistent with findings of Young et. al. [90] who conducted a methodological review focused on the search phase. In their analysis of Campbell Systematic Reviews, they found little use of automation beyond the application of ML‐embedded search engines. Collectively, these findings likely reflect broader challenges in applying ML to tasks that require more complex semantic interpretation. Rules‐based deduplication was reported in ~17% of studies. Although tools like DistillerSR [274] and Rayyan [84] began offering ML‐based deduplication in 2022–2023, no studies explicitly reported using these features, suggesting either underreporting or lagging adoption. Although ~14% of studies reported using data extraction software tools beyond standardized forms, only one study explicitly used ML to automate the extraction and assessment of study characteristics—and even then, manual corrections were required due to inaccuracies in auto‐extraction. Recent developments, such as the integration of GPT‐4 into EPPI‐Reviewer [275], may improve the accuracy and reliability of automated data extraction in future evidence synthesis workflows. In the synthesis and analysis stage, automation was widely used for quantitative analysis and generating summary tables (e.g., GRADEpro), but ML integration was limited to one study that reported using ML‐based clustering techniques to support synthesis.

Living reviews showed higher ML integration (~15%) relative to the overall data set (~5%), likely due to their continuous update requirements. However, only ~4% of review updates incorporated ML, indicating inconsistent uptake across review cycles. Tools like EPPI‐OpenAlex [86, 87, 121], which supports ongoing surveillance and citation searching, were only reported in a few cases, but offer promising avenues for future work.

#### 4.1.2 RQ 2. What Types of Automation and ML‐Based Techniques Are Currently Employed at Each Review Stage, and to What Extent Are Their Implementation Details Transparently Reported in Published Studies?

Reported automation techniques ranged from simple rules‐based methods (e.g., Boolean operators, keyword deduplication) to more sophisticated ML‐based tools, though ML use remained concentrated in screening. Active learning approaches for record prioritization and auto‐exclusion were the most commonly reported ML technique utilized. However, even among the studies that explicitly reported ML integration in screening, few described how the ML functionality was used, and fewer still detailed aspects such as model type, training data, or validation procedures. Most studies reporting use of ML‐enabled tools (~90%) did not clarify whether the optional ML functionalities were utilized. Documentation of stopping criteria in ML‐assisted screening was also limited. Among the ~83% of ML‐screening studies that applied auto‐exclusion, ~60% did not specify how or when screening was stopped, consistent with previous findings reported by König et al [53]. Of those that did report stopping methods, recall‐based criteria were most common. The lack of transparency in stopping rules impedes the evaluation of recall performance and reproducibility [53, 184].

In the search stage, ~31% of studies used supplemental tools like Google or Google Scholar, but only ~31% of those provided sufficient methodological detail to promote reproducibility, indicating a general lack of transparency in search automation. Similarly, translation tools, such as Google Translate, were used in ~4% of studies, but in ~19% of studies, translation methods (human or electronic translation) were not reported in the methods sections. Instead, they were sometimes mentioned in acknowledgments or study‐level supporting materials. These patterns suggest that, when automation is used, implementation is often described superficially. This lack of transparency is particularly problematic for translation, where tool accuracy varies by language and content and where misinterpretation of non‐English studies may introduce bias [37, 256, 257].

#### 4.1.3 RQ 3. What Justifications or Motivations Do Researchers Provide for Their Decisions to Use or Not Use ML or Other Automation Techniques in Evidence Synthesis?

Most studies using automation or ML did not provide a clear rationale. Among those that did, common motivators included reducing workload, managing large search yields, and improving efficiency. A few studies noted the utility of ML in living or rapid reviews, where time constraints made automation more attractive, and some studies cited barriers such as small sample sizes, software limitations, or inaccessible file formats that hindered automation use.

A key barrier appears to be user unawareness of ML features embedded in common tools. Many ML‐enabled tools do not clearly indicate whether ML is active, and prior work has shown that lack of awareness and training are key obstacles to adoption [276]. Our analysis lends support to this; specifically, ~90% of studies using ML‐enabled tools did not state whether ML was activated. This points to a broader issue of underreporting and the absence of transparency in software documentation and user interfaces, which collectively hinder accurate assessment of automation use [53].

### 4.2 Role of Reporting Standards

The inconsistent reporting of ML functionality, lack of implementation detail, and absence of clearly described stopping criteria observed in this review underscore a critical need for stronger guidance on documenting the use of automation in evidence synthesis. Among the major reporting standards, PRISMA 2020 [20] provides the most comprehensive guidance, recommending that authors report the software or classifier used, describe training and validation procedures, and clarify whether automation was applied to screening, search development, or data extraction. However, in practice, these recommendations were infrequently followed in the studies we reviewed. Other standards, including MECCIR [21] and ROSES [22] offer only limited guidance on what to report. Similarly, while the Cochrane Handbook [70] provides information on automation options, Cochrane has retired its standalone reporting standards and now endorses PRISMA. Despite this endorsement, many Cochrane reviews in our data set did not fully adhere to PRISMA's automation‐related recommendations. These findings highlight a clear disconnect between best‐practice reporting guidelines and real‐world practice. Addressing this gap is essential to improving transparency, interpretability, and reproducibility in evidence synthesis workflows that incorporate automation.

### 4.3 Opportunities and Challenges for AI Integration in Evidence Synthesis

While the adoption of ML in evidence synthesis remains limited, the potential benefits of AI‐assisted workflows are considerable [30, 32, 36, 46]. Automation has demonstrated value in reducing time and effort [36, 63, 64], particularly during the screening phase, and advances in NLP may extend these benefits to data extraction, synthesis, and risk of bias assessments [37]. Transformer‐based models, including BERT and GPT‐4, are particularly promising because they enable systems to interpret language in context, potentially improving the accuracy of complex tasks that traditionally require human reasoning [34, 50, 277]. However, despite these advancements, such tools have yet to be widely adopted in evidence synthesis, and concerns persist about their transparency, reliability, and integration into existing review workflows [36, 46, 50, 51, 65].

The overall rate of ML adoption across the studies analyzed in this study remains low, likely reflecting a combination of systemic, methodological, and practical factors. First, journals may be cautious about endorsing relatively new or opaque technologies without strong validation evidence. This editorial conservatism may discourage authors from using or reporting ML‐based approaches [278]. Second, while reporting standards like PRISMA 2020 [20] have begun to address automation, there is still limited formal guidance on how to appropriately integrate, validate, and document ML in evidence syntheses. Without clear expectations, researchers may be unsure how to implement ML in a methodologically sound manner or may omit it from reporting altogether. Additionally, concerns about reproducibility [52], especially when using proprietary tools, and a lack of trust in new automation technologies [279], likely inhibit adoption. Until there is greater standardization in how ML tools are evaluated, documented, and interpreted, many review teams may reasonably prefer to rely on established manual or rules‐based methods.

A central challenge is lack of user awareness regarding ML features embedded in evidence synthesis tools [276]. In our data set, the majority of studies using ML‐enabled tools did not specify whether ML functionalities were active. This ambiguity is exacerbated by the proprietary nature of many tools, which often function as “black boxes,” providing little to no information on how relevance scores are generated or how inclusion decisions are made. As a result, users may be unknowingly relying on ML without the ability to assess or mitigate potential biases. Moreover, many researchers lack formal training in ML or AI, leading to uncertainty about implementation, evaluation, and trustworthiness of these tools [279]. This skills gap likely contributes to both underutilization of ML functionality and superficial reporting when ML is used.

Despite these challenges, opportunities exist to improve the transparency and utility of AI tools in evidence synthesis. Reinforcing existing reporting standards—such as PRISMA [20], MECCIR [21], and ROSES [22]—with clearer expectations around ML use, model training, and stopping rules could help standardize documentation and support reproducibility. The development of open‐source, interpretable AI tools could also reduce dependency on proprietary systems and foster broader trust in automation. For example, integrating explainable AI techniques such as SHAP (SHapley Additive exPlanation) values or attention visualizations into evidence synthesis software could help users understand how models are making predictions and improve confidence in their decisions [280, 281].

Living reviews represent a particularly strong use case for AI integration. These continuously updated reviews require ongoing literature surveillance and reanalysis, making them well‐suited to automation. Tools like EPPI‐OpenAlex demonstrate how ML can support dynamic updating by identifying and flagging new, potentially relevant studies in real time [86, 87, 121]. As the volume of scientific literature continues to grow, such tools can help reduce reviewer burden while maintaining methodological rigor. While our findings show higher ML adoption in living reviews (~15%) compared to the overall data set (~5%), most living reviews still did not incorporate ML, indicating that the automation potential in this context remains underutilized.

Similarly, incorporating automation and AI into large‐scale reviews, such as evidence and gap maps, could significantly enhance efficiency and the timeliness of these decision‐making tools. Our findings indicate that, on average, evidence and gap maps involve screening 210% more records than systematic reviews, resulting in a considerably greater time investment. Furthermore, evidence and gap maps are 6.5 times more likely to employ single screening or automatic exclusion of records, demonstrating a higher tolerance for lower recall in these review methodologies. By normalizing the use of automation and AI in these reviews through incorporation into guidelines and standards of practice, their adoption could be promoted and barriers related to trust and implementation uncertainty reduced.

Another practical challenge involves the accessibility of full‐text documents for automated processing. As identified in one study, some articles were formatted as image‐based PDFs or used nonstandard text encoding, rendering them unreadable by ML and natural language processing tools [233]. This limitation not only impedes automation during tasks like screening and data extraction but also risks introducing unintentional exclusion of studies that cannot be processed digitally. Addressing this issue may require coordinated efforts across publishers, software developers, and review teams to promote accessible digital standards and infrastructure.

Although our review spans a critical period in the evolution of automation in evidence synthesis, it captures only the very beginning of the generative AI era. None of the studies in our data set reported using Large Language Models (LLMs) such as ChatGPT, which only became widely accessible in the final 2 years of our inclusion window. Thus, our findings likely underestimate the impact that LLMs may have on future evidence synthesis workflows. Given the typical lag between tool adoption and publication, we anticipate a notable increase in LLM use in the coming years, particularly for tasks like screening and data extraction. By taking a longer‐range retrospective view, this study provides a valuable baseline for understanding how AI integration has evolved over time and for assessing future shifts as LLMs and other advanced automation technologies become more embedded in review practices.

Finally, while this review offers insight into current trends, it also highlights a crucial gap: few studies have directly compared ML‐assisted workflows to traditional methods in terms of accuracy, efficiency, or reviewer burden. Without such evaluations, it is difficult to assess whether the theoretical benefits of automation translate into practical improvements. As evidence synthesis grows in scale and complexity, the development of rigorous comparative studies and validated evaluation frameworks will be increasingly essential to guide responsible ML adoption. With proper safeguards such as rigorous validation studies demonstrating classifier performance, clear documentation of model architecture and training data, transparency in algorithmic decision‑making (e.g., explainable AI techniques), audit mechanisms for outputs, and ongoing human oversight, alongside transparent reporting and user training, ML technologies offer promise for meeting the increasing demands of evidence synthesis. Establishing these safeguards is essential for ensuring that automation enhances rather than undermines the rigor and transparency of evidence synthesis.

### 4.4 Limitations of This Study

This study has several limitations that should be considered when interpreting the findings. First, duplicate data extraction was conducted on only ~5% of included studies; while Cohen's kappa indicated high agreement, the possibility of data extraction errors cannot be ruled out. Relatedly, a keyword highlighting strategy was used to expedite the data extraction process. While this technique helped ensure consistency in tool identification, it may have biased extraction toward commonly used technologies and led to missed detections of less familiar or inconsistently described tools.

Another limitation lies in the classification of ML‐enabled tools. Tools were categorized based on their capabilities as of the year of the study's publication. However, in many cases, it is likely that the actual review was conducted before the integration of ML functionalities into those tools. This temporal lag could result in an overestimation of ML‐enabled tool usage, particularly in the years immediately following major software updates. To mitigate this issue, the classification of tools was supplemented by external documentation to determine the timing and nature of ML feature releases. Nonetheless, this process is inherently limited by the availability and clarity of such documentation. Given the frequent versioning and rapid development of review software, outdated or ambiguous tool descriptions may still have impacted classification accuracy.

Compounding these challenges, many studies failed to specify whether ML features within ML‐enabled tools were actively used. As a result, some cases of automation may have been misclassified, particularly where ML usage was not clearly reported. To reduce inconsistencies in data extraction, the review team conducted duplicate extractions on a subset of studies, discussed discrepancies as a group, and used consensus to refine the data extraction process. Although not all studies were subjected to duplicate data extraction, this team‐based calibration likely improved consistency across the larger data set.

The scope of the review was also limited to three sources: *Cochrane Database of Systematic Reviews*, and the journals *Campbell Systematic Reviews*, and *Environmental Evidence*. While these platforms are known for methodological rigor and represent a wide range of disciplines, they do not capture the full breadth of evidence synthesis practices across other fields. As such, our findings are not likely generalizable to evidence syntheses published in outlets with different methodological standards or automation practices.

Finally, this study was designed to describe trends in the adoption and reporting of automation, including ML integration, in evidence synthesis. It does not evaluate the effectiveness, accuracy, or efficiency of these methods. As the field moves toward more widespread integration of AI and ML tools, future work is needed to rigorously compare automated approaches with traditional workflows and develop validated frameworks for performance evaluation.

## 5 Conclusions

This review examined 2271 evidence syntheses published between 2017 and 2024 in the *Cochrane Database of Systematic Reviews*, *Campbell Systematic Reviews*, and *Environmental Evidence* to assess how automation and ML are being used and reported across the evidence synthesis process. Despite increasing interest in AI technologies, their actual integration into evidence synthesis workflows remains limited. Only ~5% of studies explicitly reported using ML tools, and this usage was overwhelmingly concentrated in the screening phase. Even among studies using ML‐enabled software, few clarified whether ML functionalities were activated, suggesting significant underreporting or limited awareness of embedded AI features.

The use of automation in other stages—such as search, data extraction, and synthesis—was far less common and often relied on rule‐based tools rather than ML. The findings also reveal substantial inconsistencies in how automation is reported, with many studies omitting critical implementation details such as stopping criteria, model validation, or rationale for tool selection. These gaps impede transparency and hinder efforts to evaluate the performance and reproducibility of automated approaches.

Stronger adherence to reporting standards such as PRISMA, along with clearer guidance on documenting automation and ML use, is urgently needed to support responsible AI adoption in evidence synthesis. Moreover, user education and training are essential to ensure that researchers understand when and how ML is being used within commonly adopted tools. As the volume and complexity of research outputs continue to grow, the integration of ML‐driven workflows—if thoughtfully implemented—could help alleviate reviewer burden and accelerate review timelines, while maintaining methodological rigor.

## Author Contributions

**Kristen L. Scotti:** conceptualization, investigation, writing – original draft; methodology, visualization, writing – review and editing, formal analysis, data curation. **Sarah Young:** conceptualization, investigation, writing – review and editing, methodology, formal analysis. **Melanie A. Gainey:** formal analysis, methodology, writing – review and editing, investigation, conceptualization. **Haoyong Lan:** investigation, writing – review and editing, methodology.

## Conflicts of Interest

The authors declare no conflicts of interest.

## Supporting information

AI and Automation in Evidence Synthesis Supplemental.

## Data Availability

The data that support the findings of this study are openly available in OSF at https://osf.io/gch5e/.
