# Supplementary material for: Artificial Intelligence and Automation in Evidence Synthesis: An Investigation of Methods Employed in Cochrane, Campbell Collaboration, and Environmental Evidence Reviews
Source: Cochrane Evid Synth Methods. 2025 Aug 28;3(5):e70046. doi: 10.1002/cesm.70046 (PMC12407283; doi:10.1002/cesm.70046)
Supplement: Supplementary file 1 — AI and Automation in Evidence Synthesis Supplemental. [file CESM-3-e70046-s001.docx]

**SUPPLEMENTAL MATERIALS**

**Glossary of automation terms**

**Algorithm:** A precise, step‑by‑step set of instructions or rules that a computer follows to perform a task or solve a problem.

**Automation**: Any computer‑driven approach that replaces human effort in evidence synthesis tasks, including rule‑based algorithms and artificial intelligence/machine learning methods.

**Active Learning**: A machine‑learning strategy where the model identifies the most informative unlabeled examples and asks a human to label them, reducing the total labeling effort needed to reach high accuracy..

**Artificial Intelligence** (AI): Computer systems performing tasks that typically require human intelligence, such as reasoning or pattern recognition.

**Auto-Exclusions**: The automated removal of records deemed irrelevant by a machine learning model without human review.

**Bidirectional Encoder Representations from Transformers** (BERT): A language model that learns each word’s meaning by looking at both the words before and after it at the same time.

**Boolean Search**: A rules‑based search strategy using logical operators (AND, OR, NOT) to combine keywords.

**Classifier**: A machine learning model or algorithm trained to assign input data to one of several predefined classes or categories.

**Clustering**: An unsupervised machine learning algorithm applied to group similar data points into clusters.

**Convolutional Neural Network** (CNN): A type of deep neural network designed for processing grid-like data (such as images). It uses convolutional layers that apply learned filters (kernels) across the input to automatically extract and detect spatial hierarchies of features, followed by pooling layers to reduce dimensionality and fully connected layers for final prediction.

**Data-Driven Heuristic Stopping**: A stopping rule that adapts during an iterative process based on observed data characteristics, such as the number of consecutive non-relevant records.

**Deep Learning**: A subset of machine learning that uses neural networks (e.g., RNNs, CNNs, transformers) to capture complex data patterns.

**Electronic Translation**: The use of software tools (*e.g*., Google Translate, DeepL) to convert text from one language to another automatically.

**Feature Extraction**: The process of transforming raw data into numerical features that can be used for ML models, such as term frequency vectors or embeddings.

**Fixed Heuristic Stopping**: A stopping rule set before an iterative process begins, using a predetermined threshold or count without adaptation during execution.

**Graph-Based Methods**: Techniques that analyze data represented as graphs, which are collections of nodes (entities) connected by edges (relationships). These methods fall into two broad categories: (i) machine learning and non-machine learning methods. Machine learning methods use statistical models to learn from graph structure and features. For example, they can predict new links between nodes, classify nodes based on their connectivity patterns, or generate embeddings that capture relational context. Non‑machine learning graph algorithms apply deterministic or heuristic graph computations without learning from data. Examples include bibliographic coupling (measuring similarity by shared references) and co‑citation analysis (linking documents cited together), which define relationships purely through structure.

**Heuristic Stopping Rule**: A simple, rule-based criterion for terminating an iterative process (such as model training or screening) based on predefined conditions (e.g., a fixed number of consecutive non-informative results).

**Hybrid Approaches**: Methods combining machine learning algorithms with rule-based processes and human oversight to enhance accuracy and reliability.

**Latent Dirichlet Allocation** (LDA): A generative probabilistic model for topic modeling that represents documents as mixtures of topics and topics as mixtures of words.

**Machine Learning** (ML): A subset of artificial intelligence where systems learn predictive models from data rather than relying on predefined rules.

**Manual**: Evidence synthesis methods involving no automation or ML, often relying on spreadsheets or paper-based processes.

**ML-Assisted Screening**: The use of machine learning algorithms to support the screening process, including prioritizing records or auto-excluding studies unlikely to meet inclusion criteria.

**ML-Embedded Tool**: Software in which machine learning is a core, non‑optional component of its functionality.

**ML-Enabled Tool**: Software with optional machine learning features that users can enable or disable.

**Named Entity Recognition** (NER): A natural language processing task that identifies and classifies proper nouns in text (*e.g*., people, organizations, dates).

**Natural Language Processing** (NLP): A branch of artificial intelligence/machine learning focused on the interaction between computers and human language, enabling tasks like Named-Entity Recognition (NER) and text mining.

**Prioritized Screening:** A screening approach in which records are ranked by relevance (often *via* machine learning) so that the most likely relevant studies are reviewed first.

**Recall-Based Stopping**: A stopping criterion that ends an iterative selection process once a predefined recall level (proportion of true positives identified) is estimated to have been reached.

**Recurrent Neural Network** (RNN): A type of artificial neural network designed to handle sequential data. It maintains an internal “hidden” state that gets updated at each step of the sequence, letting it remember information from previous inputs and learn temporal patterns.

**Resource-Constrained Stopping**: A stopping criterion based on practical limitations, such as time or budget, rather than model performance.

**Rules-Based Automation**: Deterministic methods applying predefined heuristics or algorithms (e.g., deduplication by metadata).

**Supervised Learning**: A machine learning paradigm in which models are trained on labeled datasets, learning to predict outcomes or classify inputs based on example input-output pairs.

**Support Vector Machine** (SVM): A supervised machine learning algorithm that classifies data by finding optimal boundaries in feature space.

**Text Mining**: The use of algorithms to automate the process of uncovering patterns and insights from unstructured text data.

**Topic Modeling**: Unsupervised techniques for discovering abstract topics within a collection of documents, often using models like Latent Dirichlet Allocation.

**Transformer Model** (e.g., BERT): A deep learning architecture that processes entire sequences of text simultaneously, capturing contextual relationships.

**Trend-Based Stopping**: A stopping criterion that uses observed trends in data, such as a plateau in performance metrics or diminishing returns, to decide when to terminate an iterative process.

Table S-1. Summary of the current coverage of key reporting standards in respect to the use of automation, machine learning and AI tools and methods.

| **Standard Name** | **Most recent update** | **Search** | **Screening** | **Data extraction** | **Risk of Bias and Quality Assessment** | **Other** |
| --- | --- | --- | --- | --- | --- | --- |
| Preferred Reporting Items for Systematic reviews and Meta-Analysis [[1]](https://www.zotero.org/google-docs/?3be8Gc) | 2020 | "If natural language processing or text frequency analysis tools were used to identify or refine keywords, synonyms or subject indexing terms to use in the  search strategy, specify the tool(s) used."    "If a tool was used to automatically translate search strings for one database to another, specify the tool used." | "Report how automation tools were integrated within the overall study selection process."    "If an externally derived machine learning classifier was applied (e.g. Cochrane RCT Classifier), either to eliminate records or to replace a single screener, include a reference or URL to the version used. If the classifier was used to eliminate records before screening, report the number eliminated in the PRISMA flow diagram as ‘Records marked as ineligible by automation tools’."    "If an internally derived machine learning classifier was used to assist with the screening process, identify the software/classifier and version, describe how it was used (e.g. to remove records or replace a single screener) and trained (if relevant), and what internal or external validation was done to understand the risk of missed studies or incorrect classifications.”    "If machine learning algorithms were used to prioritise screening (whereby unscreened records are continually re-ordered based on screening decisions), state the software used and provide details of any screening rules applied." | "If any automation tools were used to collect data, report how the tool was used, how the tool was trained, and what internal or external validation was done to understand the risk of incorrect extractions."    "If any software was used to extract data from figures, specify the software used." | "If an automation tool was used to assess risk of bias, report how the automation tool was used, how the tool was trained, and details on the tool’s performance and internal validation."    "If an automation tool was used to support the assessment of certainty, report how the automation tool was used, how the tool was trained, and details on  the tool’s performance and internal validation." | "If abstracts or articles required translation into another language to determine their eligibility, report how these were translated." |
| Methodological Expectations of Campbell Collaboration Intervention Reviews [[2]](https://www.zotero.org/google-docs/?VWAlAE) | 2024 | None | "The list of excluded studies should list those studies which appear to meet inclusion criteria, that is, “near misses” and provide justification for exclusion. If automation is used (e.g., machine learning. AI screening of title and abstract), describe how, which software, including any validation (e.g., 10% review by human), if used." | "Describe data coding and critical appraisal process (e.g., who and how many are involved, use of pre-tested forms, machine learning, etc.) and how these are designed to minimize bias" | None | None |
| Reporting Standards for Systematic Evidence Syntheses (ROSES) [[3]](https://www.zotero.org/google-docs/?R6HeV6) | 2017 | None | None | None | None | None |

Table S-2. Classification of tools used in included studies, based on their automation and machine learning (ML) capabilities. Classifications were used to distinguish between tools that offer machine learning capabilities and those that depend on machine learning by design; these include: *Manual* (tools that involve no automation or machine learning capabilities), *Automated* (tools that apply predefined, deterministic rules or heuristics to perform tasks), *ML-enabled* (tools that integrate optional machine learning functionalities that may be disabled by the user, and *ML-embedded* (machine learning is a core, non-optimal component of the tool). For each tool, the table lists classifications by task and by publication year of the study in which it was used. Classifications reflect the tool’s capabilities as of the year of reported use. If major ML features were introduced during a study period, the classification was updated for studies published after the feature's documented release.

| **Software/Tool** | **Task** | **Study Publication Year** | **Classification** | **Justification** |
| --- | --- | --- | --- | --- |
| Abstrackr | Screening | Entire study period | ML-Enabled | Priority screening is auto-enabled in the software; users can disable [[4]](https://www.zotero.org/google-docs/?eAmtkB). Machine learning capabilities are documented as early as 2012 [[5], [6]](https://www.zotero.org/google-docs/?jb0Zif). |
| AntConc | Search query refinement | 2024 and later | ML-enabled | AntConc integrated an AI tool as an optional component by 2024 [[7]](https://www.zotero.org/google-docs/?mFHVE9). The only included study reporting use of AntConc was published in 2018, thus tool usage was classified as “automated” for all included studies. |
|  |  | Before 2024 | Automated |  |
| Article alerts | Identifying articles | Entire study period | Automated | Alerts are automated notifications triggered by new publications matching predefined keywords or search criteria. |
| Atlas.ti | Data extraction and analysis | Entire study period | Automated | ATLAS.ti is a qualitative‑data analysis suite for coding, querying and visualizing text; ML-based functionalities were introduced after the study period [[8]](https://www.zotero.org/google-docs/?jB3XuT). |
| Baidu | Translation | Entire study period | ML-embedded | Baidu employs machine learning algorithms to perform language translations [[9]](https://www.zotero.org/google-docs/?aASDaS). |
| Cadima | Screening | Entire study period | Automated | Cadima automates evidence synthesis tasks such as duplication detection and allocation of records for screening using rules-based methods [[10]](https://www.zotero.org/google-docs/?NZjAUA). |
| CINeMA | Statistical computations | Entire study period | Automated | CINeMA applies rule-based algorithms for assessing confidence in network meta-analysis results [[11]](https://www.zotero.org/google-docs/?q265NJ). |
| CiteSource | Deduplication | Entire study period | Automated | CiteSource uses rules-based algorithms for duplicate detection [[12], [13]](https://www.zotero.org/google-docs/?9lof1Q). |
| Cochrane Crowd | Screening | Entire study period | Manual | Cochrane Crowd uses crowd-sourced human screeners [[14]](https://www.zotero.org/google-docs/?Qmtd3r). |
| Cochrane RCT Filter | RCT identification | 2018 and later | ML-embedded | The RCT filter uses machine learning algorithms to identify randomized control trials. This tool was introduced by Cochrane in late 2018 [[15], [16]](https://www.zotero.org/google-docs/?cYPYHW). |
| Colandr | Screening | 2018 and later | ML-enabled | Colandr integrates relevance ranking for screening which has been auto-enabled since the 2018 release [[17]](https://www.zotero.org/google-docs/?LWmNTK). The first included study that reported the use of Colandr was published in 2020, thus tool usage for all included studies was classified as “ML-enabled”. |
|  | Data extraction | Entire study period | Automated | Colandr introduced machine learning functionalities for data extraction by at least 2025 [[18]](https://www.zotero.org/google-docs/?W5b5kx). All included studies reporting the use of Colandr for data extraction were published before these functionalities were introduced, thus tool usage was classified as “automated” for all included studies. |
| Comprehensive Meta‐Analysis (CMA) | Statistical analysis | Entire study period | Automated | Comprehensive Meta‐Analysis (CMA) is a software tool that uses predefined formulas to facilitate statistical analysis for meta-analyses [[19]](https://www.zotero.org/google-docs/?ujSRb5). |
| Connected Papers | Citation searching | Entire study period | Automated | Connected Papers builds similarity graphs that show closely related articles to a seed paper using a deterministic similarity score based solely on co‑citation and bibliographic‑coupling patterns (rules-based methods) [[20], [21]](https://www.zotero.org/google-docs/?RhPiRL). |
| Covidence | Screening | 2022 and later | ML-enabled | Covidence introduced priority screening in 2022, enabled by default through the "Most Relevant" sorting option; users may change the default sorting criteria to bypass the machine learning prioritization [[22], [23], [24]](https://www.zotero.org/google-docs/?g4BbBS). |
|  |  | Before 2022 | Automated |  |
|  | RCT Classifier | 2022 and later | ML-embedded | Covidence integrated the RCT filter, which uses a machine learning algorithm to identify randomized control trials, in May 2022 [[15], [16]](https://www.zotero.org/google-docs/?IwrMw2). |
| DeepL | Translation | Entire study period | ML-embedded | DeepL is an AI-based translation software that has leveraged machine learning since the tool was introduced [[25]](https://www.zotero.org/google-docs/?XjAuXF). |
| DistillerSR | Deduplication | 2020 and later | ML-embedded | DistillerSR introduced NLP-based deduplication functionalities in 2020 [[26]](https://www.zotero.org/google-docs/?uaRnjK). |
|  |  | Before 2020 | Automated |  |
|  | Screening | Entire study period | ML-enabled [[27]](https://www.zotero.org/google-docs/?bezCUV) | DistillerSR included active learning screening prioritization in the initial product launch in 2016 [[27], [28], [29]](https://www.zotero.org/google-docs/?DenjnI). |
| Elicit | Study identification (search) | Entire study period | ML-embedded | Elicit is an AI-driven tool for identifying relevant studies, summarizing information, and extracting data [[30], [31]](https://www.zotero.org/google-docs/?Hgjorx). |
| Endnote | Deduplication | Entire study period | Automated | Endnote is a bibliographic manager that uses rules-based algorithms for identifying and removing duplicates [[32]](https://www.zotero.org/google-docs/?07XmJ5) |
| EpiData | Data extraction, analysis | Entire study period | Automated | EpiData includes validated data‑entry forms that run statistics using fixed formulas [[33]](https://www.zotero.org/google-docs/?1Iw1q5). |
| EPPI-Reviewer | Deduplication | Entire study period | Automated | EPPI-Reviewer uses rules-based algorithms for identifying and removing duplicates [[34], [35]](https://www.zotero.org/google-docs/?9vrAL8). |
|  | Screening | Entire study period | ML-enabled | Active-learning based screening prioritization has been integrated within EPPI-Reviewer throughout the study period [[36], [37]](https://www.zotero.org/google-docs/?JbwPq6). |
|  | Classification | Entire study period | ML-embedded | EPPI-Reviewer includes the following ML-based classifiers: Cochrane RCT, Economic Evaluation, Systematic Review, and Original RCT [[34], [36], [38]](https://www.zotero.org/google-docs/?fqAq2j). |
|  | Data extraction | Entire study period | Automated | During the study period, EPPI-Reviewer offered data extraction template creation, classified as an “automated” tool for data extraction. The software now allows users to enable GPT-4 for data extraction [[39]](https://www.zotero.org/google-docs/?wPrLmT). |
| EPPI-Reviewer, Mapper | Evidence and gap map generation | Entire study period | Automated | EPPI‑Reviewer’s Mapper turns the coded records into an interactive evidence‑gap map via fixed JSON‑to‑HTML plotting routines [[35]](https://www.zotero.org/google-docs/?f88rfD). |
| EPPI-Reviewer, Visualizer | Web database generation | Entire study period | Automated | EPPI‑Reviewer Visualiser creates filterable web dashboards hosted on EPPI‑Reviewer servers, rendering the database fields through predetermined visualisation routines [[35]](https://www.zotero.org/google-docs/?IV0AV3). |
| Google Translate | Translation | Entire study period | ML-embedded | Google Translate is translation software that has leveraged machine learning throughout the study period [[40]](https://www.zotero.org/google-docs/?sHOBx6). |
| Google Forms | Data extraction | Entire study period | Automated | Google Forms allows users to create structured questionnaires and collect data in a spreadsheet [[41]](https://www.zotero.org/google-docs/?hU9E0q). |
| GradePRO | Summary tables | Entire study period | Automated | GradePRO auto-generates summary tables based on manually coded data [[42]](https://www.zotero.org/google-docs/?OVp8S8). |
| Inciteful | Search, study identification | Entire study period | Automated | Inciteful builds a custom citation graph around one or more seed papers and shows PageRank‑scored or “similar” papers using rules-based methods [[43], [44], [45]](https://www.zotero.org/google-docs/?dH5o9Z). |
| Mendeley | Deduplication | Entire study period | Automated | Mendeley uses rules-based algorithms for identifying and removing duplicates [[46]](https://www.zotero.org/google-docs/?tmCup6). |
| Microsoft Academic via EPPI | Search, updating | Entire study period | ML-embedded | EPPI leverages machine learning to refine search results and analyze data from the Microsoft Academic dataset [[47]](https://www.zotero.org/google-docs/?TXSKFz). |
| NVivo | Data extraction | Entire study period | Automatic | NVivo is a qualitative‑data‑analysis suite for coding, querying, and visualizing text [[48]](https://www.zotero.org/google-docs/?4TAznd). NVivo version 15 (2024) integrated the “Lumivero AI Assistant” [[49]](https://www.zotero.org/google-docs/?Yw2hwJ), but all included studies describing use of this software were published before this integration. |
| OpenAlex via EPPI | Search, updating | Entire study period | ML-embedded | EPPI leverages machine learning to refine search results and analyze data from the Open Alex dataset [[47]](https://www.zotero.org/google-docs/?c3SbiX). |
| Paperfetcher | Citation searching | Entire study period | Automated | Paperfetcher uses rules-based methods for automated citation searching [[50]](https://www.zotero.org/google-docs/?WaM0ZP). |
| Publish or Perish | Bibliographic importing | Entire study period | Automated | Publish or Perish retrieves citation data from sources like Google Scholar and converts them into bibliometric indicators using fixed formulae [[51]](https://www.zotero.org/google-docs/?z2Qekp). |
| PubMed Related Citations | Citation searching | Entire study period | Automated | PubMed’s Related Citations feature returns a ranked list of papers whose titles, abstracts and MeSH terms are most similar to a seed article, calculated using a similarity formula [[52]](https://www.zotero.org/google-docs/?xCXpdP). |
| PubReMiner | Keyword/query optimization | Entire study period | Automated | PubReMiner is a web-based tool designed to enhance PubMed searches by analyzing search results and generating frequency tables; operations are based on statistical analyses of term frequencies, relying on manual input and user interaction for query optimization [[53]](https://www.zotero.org/google-docs/?qTwp30). |
| Qualtrics Forms | Data extraction | Entire study period | Automated | Qualtrics Forms facilitates building data‑extraction sheets where flow is controlled by fixed rules [[54]](https://www.zotero.org/google-docs/?mpDAv9). |
| R, find_duplicates | Deduplication | Entire study period | Automated | The R “find duplicates” function uses rules-based methods for detecting duplicates [[55]](https://www.zotero.org/google-docs/?HO2lOY). |
| R, greylitsearcher | Search | Entire study period | Automated | The R, “greylitsearcher” package uses a rules-based algorithm to access Google's site search functionality [[56]](https://www.zotero.org/google-docs/?OXC9g4). |
| R, citationtracer | Citation searching | Entire study period | Automated | The R, “citationtracer” package accesses Lens.org for forward and backward citation searching [[57]](https://www.zotero.org/google-docs/?FyOU3J). |
| R, metaDigitise | Digitization | Entire study period | Automated | The R, “metaDigitise” package is designed to facilitate the extraction of data from figures using rules-based methods [[58]](https://www.zotero.org/google-docs/?aEFz25). |
| R, metafor | Statistical analysis | Entire study period | Automated | The R, “metafor” package provides tools for fitting fixed-effects, random-effects, and mixed-effects models to meta-analytic data using defined formulas [[59]](https://www.zotero.org/google-docs/?t7w640). |
| R, clubSandwich | Statistical analysis | Entire study period | Automated | The R, “clubSandwich” package performs robust variance estimation (RVE) calculations [[60]](https://www.zotero.org/google-docs/?mp6Sv3). |
| R, wordcloud | Search keyword optimization | Entire study period | Automated | The wordcloud package in R turns a word‑frequency table into a visualization where each word’s font size reflects its count, using deterministic algorithms [[61]](https://www.zotero.org/google-docs/?2Zrqfr). |
| R, Robumeta | Statistical analysis | Entire study period | Automated | The R, “robumeta” package performs robust‑variance meta‑regression, implementing the Hedges‑Tipton statistical formulas to analyse correlated effect sizes [[62]](https://www.zotero.org/google-docs/?6MFUz4). |
| R, robvis | Bias assessment | Entire study period | Automated | The R, “robovis” package converts risk‑of‑bias assessment tables into traffic‑light and summary bar plots using fixed plotting routines [[63]](https://www.zotero.org/google-docs/?4BVmxK). |
| Rayyan | Deduplication | 2023 and later | ML-embedded | Rayyan introduced ML-powered deduplication features in 2023 [[64]](https://www.zotero.org/google-docs/?R3N47B). |
|  |  | Before 2023 | Automated |  |
|  | Screening | 2020 and later | ML-enabled | Rayyan is a systematic review software that integrated active-learning based prioritized screening by 2020 [[65], [66], [67]](https://www.zotero.org/google-docs/?S8V5BO); reported tool usage prior to 2020 was classified as “automated.” |
|  |  | Before 2020 | Automated |  |
| RedCap | Data extraction | Entire study period | Automated | REDCap is a web‑based platform for designing and managing databases via user‑defined forms and validation rules [[68]](https://www.zotero.org/google-docs/?kKNpr9). |
| RefWorks | Deduplication | Entire study period | Automated | RefWorks is a bibliographic manager that uses rules-based algorithms for identifying and removing duplicates [[69]](https://www.zotero.org/google-docs/?3YjabB). |
| RevMan | Data extraction | Entire study period | Automated | RevMan is Cochrane's software for preparing and maintaining systematic reviews and meta-analyses. It offers automation tools for data management, statistical analysis, and reporting [[70]](https://www.zotero.org/google-docs/?DudGMT). |
|  | Statistical analysis |  |  |  |
| Robot Reviewer | Classification | Entire study period | ML-embedded | Robot Reviewer includes ML-based classification and data extraction tools, including the capabilities for identifying PICO characteristics of studies [[71]](https://www.zotero.org/google-docs/?RmYBNa). |
|  | Data extraction |  |  |  |
| Screen4Me | Screening | Classified based on components utilized [[72]](https://www.zotero.org/google-docs/?lHE3RW) (see Cochrane Crowd, RCT filter, and Covidence) | | |
| SpiderCite | Citation searching | Entire study period | Automated | SpiderCite facilitates forward and backward citation searching and uses Lens.org data to automate searching; the process is deterministic [[73]](https://www.zotero.org/google-docs/?aBz2bF). |
| Spreadsheet | Screening | Entire study period | Manual | Spreadsheets (e.g., Excel) are used for manually tracking screening decisions in evidence synthesis workflows. |
| SPSS | Statistical analysis | Entire study period | Automated | IBM SPSS Statistics is point‑and‑click software suite for performing descriptive statistics [[74]](https://www.zotero.org/google-docs/?thc2yx). |
| Stata | Statistical analysis | Entire study period | Automated | Stata is a comprehensive statistical and data‑science environment for cleaning, visualising and modelling data. While the software currently integrates ML modeling functionality, included studies reporting usage described basic statistical calculations [[75]](https://www.zotero.org/google-docs/?T0qRV7). |
| Swift Active Screener | Screening | Entire study period | ML-enabled | SWIFT‑Active Screener is a web‑based tool that uses active‑learning machine‑learning models to continuously rank and prioritize citations during title‑and‑abstract screening [[76]](https://www.zotero.org/google-docs/?Ki2Va9). |
| Sysrev | Screening | Entire study period | ML-enabled | Sysrev is a web platform for screening and extracting data that utilizes active‑learning models trained on reviewers’ decisions [[77]](https://www.zotero.org/google-docs/?K4DhbV). |
| SysReview | Review management software | Entire study period | Automated | SysReview supports bibliographic record imports, deduplication and tracking of records and decisions in evidence synthesis workflows; it operates with user‑defined rules [[78]](https://www.zotero.org/google-docs/?dEJyFo). |
| TerMine | Search query refinement | Entire study period | Automated | TerMine generates a ranked list of “interesting” terms using rules-based methods [[79]](https://www.zotero.org/google-docs/?gduT0A). |
| VOSViewer | Bibliometric network construction | Entire study period | Automated | VOSViewer constructs and visualizes bibliometric networks (co‑authorship, citation, keyword co‑occurrence, etc.) using deterministic similarity‑based mapping and modularity‑style clustering algorithms [[80]](https://www.zotero.org/google-docs/?s9lj2n). |
| WebPlotDigitizer | Data extraction from figures | 2024 and later | ML-embedded | WebPlotDigitizer is digitization software that enables data extraction from figures. Version 5 (2024) combines multimodal machine learning methods, traditional computer vision algorithms, and manual tools [[81]](https://www.zotero.org/google-docs/?O4B8GC). No included studies reported usage of WebPlotDigitizer after the introduction of ML capabilities, thus this tool was classified as “automated” for all included studies reporting usage. |
|  |  | Before 2024 | Automated |  |
| Yale MeSH Analyzer | Search query refinement | Entire study period | Automated | Yale MeSH Analyzer automatically pulls MeSH headings and related PubMed metadata for a set of PMIDs to build a grid that can be used to refine search strategies, and it is a rule‑based tool [[82]](https://www.zotero.org/google-docs/?aWXK0q) |
| Yandex | Translation | Entire study period | ML-embedded | Yandex is a software translation tool that uses neural‑machine‑translation models to translate text [[83]](https://www.zotero.org/google-docs/?NF5Cwv). |
| Zotero | Deduplication | Entire study period | Automated | Zotero is a bibliographic manager that uses rules-based algorithms for identifying and removing duplicates [[84]](https://www.zotero.org/google-docs/?ngdWQq). |

**References**

[[1] M. J. Page *et al.*, “The PRISMA 2020 statement: an updated guideline for reporting systematic reviews,” *BMJ*, vol. 372, p. n71, Mar. 2021, doi: 10.1136/bmj.n71.](https://www.zotero.org/google-docs/?YtEfxD)

[[2] C. Collaboration, “Methodological expectations of Campbell Collaboration intervention reviews (MECCIR),” *Oslo Nor.*, 2024.](https://www.zotero.org/google-docs/?YtEfxD)

[[3] N. R. Haddaway, B. Macura, P. Whaley, and A. S. Pullin, “ROSES RepOrting standards for Systematic Evidence Syntheses: pro forma, flow-diagram and descriptive summary of the plan and conduct of environmental systematic reviews and systematic maps,” *Environ. Evid.*, vol. 7, no. 1, p. 7, Mar. 2018, doi: 10.1186/s13750-018-0121-7.](https://www.zotero.org/google-docs/?YtEfxD)

[[4] J. Starks, “KTDRR and Campbell Collaboration Research Evidence Training:  Management/Analysis Tools for Reviews - Abstrackr.” Accessed: Feb. 05, 2025. [Online]. Available: https://ktdrr.org/training/webcasts/webcast67/index.html](https://www.zotero.org/google-docs/?YtEfxD)

[[5] B. C. Wallace, K. Small, C. E. Brodley, J. Lau, and T. A. Trikalinos, “Deploying an interactive machine learning system in an evidence-based practice center: abstrackr,” in *Proceedings of the 2nd ACM SIGHIT International Health Informatics Symposium*, in IHI ’12. New York, NY, USA: Association for Computing Machinery, Jan. 2012, pp. 819–824. doi: 10.1145/2110363.2110464.](https://www.zotero.org/google-docs/?YtEfxD)

[[6] J. Rathbone, T. Hoffmann, and P. Glasziou, “Faster title and abstract screening? Evaluating Abstrackr, a semi-automated online screening program for systematic reviewers,” *Syst. Rev.*, vol. 4, no. 1, p. 80, Jun. 2015, doi: 10.1186/s13643-015-0067-6.](https://www.zotero.org/google-docs/?YtEfxD)

[[7] L. Anthony, “A Comprehensive Guide to AntConc 4: New Tools, Features, and AI Integration,” Aug. 2024, Accessed: Feb. 05, 2025. [Online]. Available: https://osf.io/qzp64/](https://www.zotero.org/google-docs/?YtEfxD)

[[8] “ATLAS.ti | The #1 Software for Qualitative Data Analysis,” ATLAS.ti. Accessed: Feb. 19, 2025. [Online]. Available: https://atlasti.com](https://www.zotero.org/google-docs/?YtEfxD)

[[9] Z. He, “Baidu translate: Research and products,” in *Proceedings of the Fourth Workshop on Hybrid Approaches to Translation (HyTra)*, 2015, pp. 61–62. doi: 10.18653/v1/W15-4110.](https://www.zotero.org/google-docs/?YtEfxD)

[[10] C. Kohl *et al.*, “Online tools supporting the conduct and reporting of systematic reviews and systematic maps: a case study on CADIMA and review of existing tools,” *Environ. Evid.*, vol. 7, no. 1, p. 8, Feb. 2018, doi: 10.1186/s13750-018-0115-5.](https://www.zotero.org/google-docs/?YtEfxD)

[[11] A. Nikolakopoulou *et al.*, “CINeMA: An approach for assessing confidence in the results of a network meta-analysis,” *PLOS Med.*, vol. 17, no. 4, p. e1003082, Apr. 2020, doi: 10.1371/journal.pmed.1003082.](https://www.zotero.org/google-docs/?YtEfxD)

[[12] “Analyze the Utility of Information Sources and Retrieval Methodologies for Evidence Synthesis.” Accessed: Feb. 05, 2025. [Online]. Available: https://www.eshackathon.org/CiteSource/](https://www.zotero.org/google-docs/?YtEfxD)

[[13] *camaradesuk/ASySD*. (Jan. 28, 2025). HTML. CAMARADES. Accessed: Feb. 05, 2025. [Online]. Available: https://github.com/camaradesuk/ASySD](https://www.zotero.org/google-docs/?YtEfxD)

[[14] A. Noel-Storr, G. Dooley, L. Affengruber, and G. Gartlehner, “Citation screening using crowdsourcing and machine learning produced accurate results: evaluation of Cochrane’s modified Screen4Me service,” *J. Clin. Epidemiol.*, vol. 130, pp. 23–31, 2021.](https://www.zotero.org/google-docs/?YtEfxD)

[[15] J. Thomas *et al.*, “Machine learning reduced workload with minimal risk of missing studies: development and evaluation of a randomized controlled trial classifier for Cochrane Reviews,” *J. Clin. Epidemiol.*, vol. 133, pp. 140–151, May 2021, doi: 10.1016/j.jclinepi.2020.11.003.](https://www.zotero.org/google-docs/?YtEfxD)

[[16] B. C. Wallace, A. Noel-Storr, I. J. Marshall, A. M. Cohen, N. R. Smalheiser, and J. Thomas, “Identifying reports of randomized controlled trials (RCTs) via a hybrid machine learning and crowdsourcing approach,” *J. Am. Med. Inform. Assoc.*, vol. 24, no. 6, pp. 1165–1168, Nov. 2017, doi: 10.1093/jamia/ocx053.](https://www.zotero.org/google-docs/?YtEfxD)

[[17] S. h. Cheng *et al.*, “Using machine learning to advance synthesis and use of conservation and environmental evidence,” *Conserv. Biol.*, vol. 32, no. 4, pp. 762–764, 2018, doi: 10.1111/cobi.13117.](https://www.zotero.org/google-docs/?YtEfxD)

[[18] J. Library, “JABSOM Library: Colandr for Systematic Reviews: Getting Started.” Accessed: Jul. 29, 2025. [Online]. Available: https://hslib.jabsom.hawaii.edu/colandr/getting_started](https://www.zotero.org/google-docs/?YtEfxD)

[[19] “What’s New in Version 4? | Comprehensive Meta-Analysis.” Accessed: Jul. 29, 2025. [Online]. Available: https://meta-analysis.com/pages/new_v4?cart=BG2H15536093](https://www.zotero.org/google-docs/?YtEfxD)

[[20] P. K. Behera, S. J. Jain, and A. Kumar, “Visual Exploration of Literature Using Connected Papers: A Practical Approach,” *Issues Sci. Technol. Librariansh.*, no. 104, 2023, Accessed: Feb. 18, 2025. [Online]. Available: https://journals.library.ualberta.ca/istl/index.php/istl/article/view/2760](https://www.zotero.org/google-docs/?YtEfxD)

[[21] E. Smolyansky, “Connected Papers — a visual tool for researchers to find and explore academic papers,” Connected Papers. Accessed: Dec. 20, 2024. [Online]. Available: https://medium.com/connectedpapers/announcing-connected-papers-a-visual-tool-for-researchers-to-find-and-explore-academic-papers-89146a54c7d4](https://www.zotero.org/google-docs/?YtEfxD)

[[22] J. Starks, “KTDRR and Campbell Collaboration Research Evidence Training:  Management/Analysis Tools for Reviews - Covidence.” Accessed: Feb. 05, 2025. [Online]. Available: https://ktdrr.org/training/webcasts/webcast67/index.html](https://www.zotero.org/google-docs/?YtEfxD)

[[23] A. Walton, “Covidence Product Updates and Bug Fixes,” Covidence. Accessed: Feb. 10, 2025. [Online]. Available: https://www.covidence.org/blog/release-notes-december-2022-machine-learning/](https://www.zotero.org/google-docs/?YtEfxD)

[[24] “Covidence - Better systematic review management,” Covidence. Accessed: Feb. 19, 2025. [Online]. Available: https://www.covidence.org/](https://www.zotero.org/google-docs/?YtEfxD)

[[25] L. Linlin, “Artificial Intelligence Translator DeepL Translation Quality Control,” *Procedia Comput. Sci.*, vol. 247, pp. 710–717, 2024.](https://www.zotero.org/google-docs/?YtEfxD)

[[26] “What’s New in DistillerSR: Next Level Automation,” DistillerSR. Accessed: Jul. 29, 2025. [Online]. Available: https://www.distillersr.com/resources/updates/whats-new-in-distillersr-next-level-automation](https://www.zotero.org/google-docs/?YtEfxD)

[[27] C. Hamel, S. E. Kelly, K. Thavorn, D. B. Rice, G. A. Wells, and B. Hutton, “An evaluation of DistillerSR’s machine learning-based prioritization tool for title/abstract screening – impact on reviewer-relevant outcomes,” *BMC Med. Res. Methodol.*, vol. 20, no. 1, p. 256, Dec. 2020, doi: 10.1186/s12874-020-01129-1.](https://www.zotero.org/google-docs/?YtEfxD)

[[28] “No Second Screener? There’s A Robot For That,” DistillerSR. Accessed: Jul. 29, 2025. [Online]. Available: https://www.distillersr.com/resources/blog/no-second-screener-theres-a-robot-for-that](https://www.zotero.org/google-docs/?YtEfxD)

[[29] “DistillerSR AI,” DistillerSR. Accessed: Jul. 29, 2025. [Online]. Available: https://www.distillersr.com/products/distillersrai](https://www.zotero.org/google-docs/?YtEfxD)

[[30] J. Kung, “Elicit (product review),” *J. Can. Health Libr. Assoc. J. Assoc. Bibl. Santé Can.*, vol. 44, no. 1, Apr. 2023, doi: 10.29173/jchla29657.](https://www.zotero.org/google-docs/?YtEfxD)

[[31] S. Whitfield and M. A. Hofmann, “Elicit: AI literature review research assistant,” *Public Serv. Q.*, vol. 19, no. 3, pp. 201–207, Jul. 2023, doi: 10.1080/15228959.2023.2224125.](https://www.zotero.org/google-docs/?YtEfxD)

[[32] *EndNote - The Best Citation & Reference Management Tool*. Accessed: Feb. 23, 2025. [Online]. Available: https://endnote.com/](https://www.zotero.org/google-docs/?YtEfxD)

[[33] “EpiData Software - http://www.epidata.dk.” Accessed: Feb. 19, 2025. [Online]. Available: https://www.epidata.dk/](https://www.zotero.org/google-docs/?YtEfxD)

[[34] “EPPI-Reviewer: systematic review software.” Accessed: Feb. 19, 2025. [Online]. Available: https://eppi.ioe.ac.uk/cms/Default.aspx?tabid=2914](https://www.zotero.org/google-docs/?YtEfxD)

[[35] “Automation tools in EPPI-Reviewer.” Accessed: Feb. 05, 2025. [Online]. Available: https://eppi.ioe.ac.uk/cms/Default.aspx?tabid=3772&utm_source=chatgpt.com](https://www.zotero.org/google-docs/?YtEfxD)

[[36] “Machine learning functionality in EPPI-Reviewer.”](https://www.zotero.org/google-docs/?YtEfxD)

[[37] J. Starks, “KTDRR and Campbell Collaboration Research Evidence Training:  Management/Analysis Tools for Reviews – EPPI-Reviewer.” Accessed: Feb. 05, 2025. [Online]. Available: https://ktdrr.org/training/webcasts/webcast67/index.html](https://www.zotero.org/google-docs/?YtEfxD)

[[38] C. Stansfield, G. Stokes, and J. Thomas, “Applying machine classifiers to update searches: Analysis from two case studies,” *Res. Synth. Methods*, vol. 13, no. 1, pp. 121–133, Jan. 2022, doi: 10.1002/jrsm.1537.](https://www.zotero.org/google-docs/?YtEfxD)

[[39] “Automated data extraction using GPT-4.” Accessed: Feb. 10, 2025. [Online]. Available: https://eppi.ioe.ac.uk/cms/Default.aspx?tabid=3921](https://www.zotero.org/google-docs/?YtEfxD)

[[40] “Google Translate.” Accessed: Feb. 19, 2025. [Online]. Available: https://translate.google.com/?sl=auto&tl=en&op=translate](https://www.zotero.org/google-docs/?YtEfxD)

[[41] G. Workspace, “Google Forms: Online Form Builder,” Google Workspace. Accessed: Feb. 19, 2025. [Online]. Available: https://workspace.google.com/products/forms/](https://www.zotero.org/google-docs/?YtEfxD)

[[42] “GRADEpro.” Accessed: Feb. 23, 2025. [Online]. Available: https://www.gradepro.org/](https://www.zotero.org/google-docs/?YtEfxD)

[[43] S. Prasad and R. Chakravarty, “Connecting the dots: research discovery using network analysis algorithms,” *Digit. Univ. Nebraska–Lincoln Available Httpsdigitalcommons Unl Edulibphilprac6363accessed 17 January 2022*, 2021, Accessed: Feb. 11, 2025. [Online]. Available: https://www.academia.edu/download/74183472/Connecting_the_Dots.pdf](https://www.zotero.org/google-docs/?YtEfxD)

[[44] M. Weishuhn, “Inciteful: citation network exploration.” 2022.](https://www.zotero.org/google-docs/?YtEfxD)

[[45] “Paper Discovery Explained,” Inciteful Docs. Accessed: Feb. 19, 2025. [Online]. Available: https://help.inciteful.xyz/paper-disovery-explained.html](https://www.zotero.org/google-docs/?YtEfxD)

[[46] “deduplication,” Mendeley Blog. Accessed: Jul. 29, 2025. [Online]. Available: https://blog.mendeley.com/tag/deduplication/](https://www.zotero.org/google-docs/?YtEfxD)

[[47] “Using Microsoft Academic in EPPI-Reviewer Web.” Accessed: Feb. 05, 2025. [Online]. Available: https://eppi.ioe.ac.uk/cms/Default.aspx?tabid=3772&utm_source=chatgpt.com](https://www.zotero.org/google-docs/?YtEfxD)

[[48] “NVivo: Leading Qualitative Data Analysis Software,” Lumivero. Accessed: Feb. 19, 2025. [Online]. Available: https://lumivero.com/products/nvivo/](https://www.zotero.org/google-docs/?YtEfxD)

[[49] “NVivo: Leading Qualitative Data Analysis Software,” Lumivero. Accessed: Jul. 29, 2025. [Online]. Available: https://lumivero.com/products/nvivo/](https://www.zotero.org/google-docs/?YtEfxD)

[[50] A. Pallath and Q. Zhang, “*Paperfetcher*  : A tool to automate handsearching and citation searching for systematic reviews,” *Res. Synth. Methods*, vol. 14, no. 2, pp. 323–335, Mar. 2023, doi: 10.1002/jrsm.1604.](https://www.zotero.org/google-docs/?YtEfxD)

[[51] A.-W. Harzing -, “Publish or Perish,” Harzing.com. Accessed: Jul. 29, 2025. [Online]. Available: https://harzing.com/resources/publish-or-perish](https://www.zotero.org/google-docs/?YtEfxD)

[[52] “PubMed Update: Related Citation Links in Search Results.” Accessed: Jul. 29, 2025. [Online]. Available: https://www.nlm.nih.gov/pubs/techbull/tb.html](https://www.zotero.org/google-docs/?YtEfxD)

[[53] L. Slater, “PubMed PubReMiner,” *J. Can. Health Libr. Assoc. Assoc. Bibl. Santé Can.*, vol. 33, no. 2, pp. 106–107, 2012.](https://www.zotero.org/google-docs/?YtEfxD)

[[54] “Qualtrics XM - Experience Management Software,” Qualtrics. Accessed: Feb. 19, 2025. [Online]. Available: https://www.qualtrics.com/](https://www.zotero.org/google-docs/?YtEfxD)

[[55] “find_duplicates function - RDocumentation.” Accessed: Jul. 29, 2025. [Online]. Available: https://www.rdocumentation.org/packages/revtools/versions/0.4.1/topics/find_duplicates](https://www.zotero.org/google-docs/?YtEfxD)

[[56] N. Haddaway, *greylitsearcher: An R package and Shiny app for systematic and transparent searching for grey literature*. (Apr. 12, 2022). Zenodo. Accessed: Feb. 18, 2025. [Online]. Available: https://zenodo.org/records/6451616](https://www.zotero.org/google-docs/?YtEfxD)

[[57] N. Haddaway, M. Grainger, and C. Gray, *citationchaser: Perform Forward and Backwards Chasing in Evidence Syntheses*. (Jan. 27, 2022). Accessed: Feb. 18, 2025. [Online]. Available: https://cran.r-project.org/web/packages/citationchaser/index.html](https://www.zotero.org/google-docs/?YtEfxD)

[[58] J. L. Pick, S. Nakagawa, and D. W. A. Noble, “Reproducible, flexible and high-throughput data extraction from primary literature: The metaDigitise **R** package,” Jan. 15, 2018, *Cold Spring Harbor Laboratory*. doi: 10.1101/247775.](https://www.zotero.org/google-docs/?YtEfxD)

[[59] W. Viechtbauer, *metafor: Meta-Analysis Package for R*. (Jan. 28, 2025). Accessed: Jul. 29, 2025. [Online]. Available: https://cran.r-project.org/web/packages/metafor/index.html](https://www.zotero.org/google-docs/?YtEfxD)

[[60] J. E. Pustejovsky, S. Pekofsky, and J. Zhang, *clubSandwich: Cluster-Robust (Sandwich) Variance Estimators with Small-Sample Corrections*. (Apr. 01, 2025). Accessed: Jul. 29, 2025. [Online]. Available: https://cran.r-project.org/web/packages/clubSandwich/index.html](https://www.zotero.org/google-docs/?YtEfxD)

[[61] “wordcloud function - RDocumentation.” Accessed: Jul. 29, 2025. [Online]. Available: https://www.rdocumentation.org/packages/wordcloud/versions/2.6/topics/wordcloud](https://www.zotero.org/google-docs/?YtEfxD)

[[62] Z. Fisher, E. Tipton, and H. Zhipeng, *robumeta: Robust Variance Meta-Regression*. (Mar. 28, 2023). Accessed: Jul. 29, 2025. [Online]. Available: https://cran.r-project.org/web/packages/robumeta/index.html](https://www.zotero.org/google-docs/?YtEfxD)

[[63] *robvis, a visualization tool for risk-of-bias assessments*. Accessed: Jul. 29, 2025. [Online]. Available: https://cran.r-project.org/web/packages/robvis/vignettes/Introduction_to_robvis.html](https://www.zotero.org/google-docs/?YtEfxD)

[[64] “What’s New in Rayyan – January 2023,” Rayyan Blog. Accessed: Jul. 29, 2025. [Online]. Available: https://blog.rayyan.ai/2024/08/08/whats-new-in-rayyan-january-2023/](https://www.zotero.org/google-docs/?YtEfxD)

[[65] M. Ouzzani, H. Hammady, Z. Fedorowicz, and A. Elmagarmid, “Rayyan—a web and mobile app for systematic reviews,” *Syst. Rev.*, vol. 5, no. 1, p. 210, Dec. 2016, doi: 10.1186/s13643-016-0384-4.](https://www.zotero.org/google-docs/?YtEfxD)

[[66] H. M. says, “What’s New in Rayyan – January 2023,” Rayyan Blog. Accessed: Feb. 10, 2025. [Online]. Available: https://blog.rayyan.ai/2024/08/08/whats-new-in-rayyan-january-2023/](https://www.zotero.org/google-docs/?YtEfxD)

[[67] J. Starks, “KTDRR and Campbell Collaboration Research Evidence Training:  Management/Analysis Tools for Reviews - Rayyan.” Accessed: Feb. 05, 2025. [Online]. Available: https://ktdrr.org/training/webcasts/webcast67/index.html](https://www.zotero.org/google-docs/?YtEfxD)

[[68] “REDCap.” Accessed: Feb. 19, 2025. [Online]. Available: https://project-redcap.org/](https://www.zotero.org/google-docs/?YtEfxD)

[[69] K. Greene, “Guides: RefWorks Guide: View Duplicates.” Accessed: Jul. 29, 2025. [Online]. Available: https://guides.dml.georgetown.edu/c.php?g=729330&p=5457479](https://www.zotero.org/google-docs/?YtEfxD)

[[70] “RevMan Web, Cochrane’s systematic-review production software, is now available to the wider academic community | Cochrane.” Accessed: Jul. 29, 2025. [Online]. Available: https://www.cochrane.org/about-us/news/revman-web-cochranes-systematic-review-production-software-now-available-wider-academic](https://www.zotero.org/google-docs/?YtEfxD)

[[71] I. J. Marshall, J. Kuiper, E. Banner, and B. C. Wallace, “Automating biomedical evidence synthesis: RobotReviewer,” in *Proceedings of the conference. Association for Computational Linguistics. Meeting*, NIH Public Access, 2017, p. 7. doi: 10.18653/v1/P17-4002.](https://www.zotero.org/google-docs/?YtEfxD)

[[72] “‘Screen For Me’: harnessing the efficiencies of machine learning and Cochrane Crowd to identify randomized trials for Cochrane Reviews | Cochrane Colloquium Abstracts.” Accessed: Dec. 31, 2024. [Online]. Available: https://abstracts.cochrane.org/2018-edinburgh/screen-me-harnessing-efficiencies-machine-learning-and-cochrane-crowd-identify](https://www.zotero.org/google-docs/?YtEfxD)

[[73] J. Clark, P. Glasziou, C. Del Mar, A. Bannach-Brown, P. Stehlik, and A. M. Scott, “A full systematic review was completed in 2 weeks using automation tools: a case study,” *J. Clin. Epidemiol.*, vol. 121, pp. 81–90, May 2020, doi: 10.1016/j.jclinepi.2020.01.008.](https://www.zotero.org/google-docs/?YtEfxD)

[[74] “IBM SPSS Statistics.” Accessed: Jul. 29, 2025. [Online]. Available: https://www.ibm.com/products/spss-statistics](https://www.zotero.org/google-docs/?YtEfxD)

[[75] “Statistical software for data science | Stata.” Accessed: Jul. 29, 2025. [Online]. Available: https://www.stata.com/](https://www.zotero.org/google-docs/?YtEfxD)

[[76] B. E. Howard *et al.*, “SWIFT-Active Screener: Accelerated document screening through active learning and integrated recall estimation,” *Environ. Int.*, vol. 138, p. 105623, May 2020, doi: 10.1016/j.envint.2020.105623.](https://www.zotero.org/google-docs/?YtEfxD)

[[77] T. Bozada, J. Borden, J. Workman, M. Del Cid, J. Malinowski, and T. Luechtefeld, “Sysrev: A FAIR Platform for Data Curation and Systematic Evidence Review,” *Front. Artif. Intell.*, vol. 4, Aug. 2021, doi: 10.3389/frai.2021.685298.](https://www.zotero.org/google-docs/?YtEfxD)

[[78] A. Higginson and R. Neville, *SysReview*. (2014). The University of Queensland.](https://www.zotero.org/google-docs/?YtEfxD)

[[79] N. Okazaki, “Acromine Demonstration.” Accessed: Jul. 29, 2025. [Online]. Available: https://www.nactem.ac.uk/software/termine/](https://www.zotero.org/google-docs/?YtEfxD)

[[80] “VOSviewer - Visualizing scientific landscapes,” VOSviewer. Accessed: Jul. 29, 2025. [Online]. Available: https://www.vosviewer.com//](https://www.zotero.org/google-docs/?YtEfxD)

[[81] “automeris.io: Computer vision assisted data extraction from charts using WebPlotDigitizer.” Accessed: Feb. 19, 2025. [Online]. Available: https://automeris.io/](https://www.zotero.org/google-docs/?YtEfxD)

[[82] R. Hocking, “Yale MeSH Analyzer,” *J. Can. Health Libr. Assoc. Assoc. Bibl. Santé Can.*, vol. 38, no. 3, 2017, Accessed: Feb. 11, 2025. [Online]. Available: https://journals.library.ualberta.ca/jchla/index.php/jchla/article/download/29336/21388](https://www.zotero.org/google-docs/?YtEfxD)

[[83] M. van Hees, P. Kozłowska, and N. Tian, “Web-based automatic translation: the Yandex. Translate API,” 2015, Accessed: Feb. 19, 2025. [Online]. Available: https://staas.home.xs4all.nl/t/swtr/documents/wt2015_yandex_translate.pdf](https://www.zotero.org/google-docs/?YtEfxD)

[[84] K. Mueen Ahmed and B. E. A. Dhubaib, “Zotero: A bibliographic assistant to researcher,” *J. Pharmacol. Pharmacother.*, vol. 2, no. 4, pp. 304–305, 2011, doi: 10.4103/0976-500X.85940.](https://www.zotero.org/google-docs/?YtEfxD)
